# Decision-Making at End-of-Life for Children With Cancer: A Systematic Review and Meta-Bioethical Analysis

**DOI:** 10.3389/fonc.2021.739092

**Published:** 2021-10-15

**Authors:** Luis Enrique Juárez-Villegas, Myriam M. Altamirano-Bustamante, Marta M. Zapata-Tarrés

**Affiliations:** ^1^ Department of Hematology-Oncology, Hospital Infantil de Mexico Federico Gómez, Mexico City, Mexico; ^2^ Master and Doctorate Program in Medical and Health Sciences, Universidad Nacional Autónoma de México, Mexico City, Mexico; ^3^ Metabolic Diseases Research Unit, Cross-functional Bioethics Group, Centro Médico Nacional Siglo XXI, Instituto Mexicano del Seguro Social, Mexico City, Mexico; ^4^ Research Coordination, Fundación IMSS AC, Mexico City, Mexico

**Keywords:** Children with cancer, end-of-life, decision-making, ethical dilemmas, axiology, systematic review

## Abstract

**Background:**

Evidence shows that medical education includes a variety of basic and clinical skills. Ethical and human values are not typically considered in medical school curricula, and this is evident in medical practice in certain scenarios such as decision-making at pediatric cancer patients' end of life.

**Methods:**

This study explores a bioethical approach to address complex decision-making at the end of life in children and adolescents with cancer. We are a cross-functional group of scientists from several academic disciplines who conducted a systematic review of the literature using our newly developed meta-bioethical analysis and synthesis of findings. The search was carried out in five databases, resulting in 10 research papers. Following quality screening, seven articles were ultimately selected for further analysis.

**Results:**

Our focus is on the state of the art to better understand the bioethical deliberation at the end of life in pediatric oncology. Here, we report a systematic review that includes (i) classification of the screened articles by the type of decision-making they use, ii) the system values that are at the core of the decision-making at the end of life, and iii) bioethical and ethical discernment queries. We conclude with a discussion regarding the best practices of ethical discernment and decision-making at the end of life.

This study highlights the need to develop more research to better understand the influence and origin of these multidimensional factors determining critical decisions that define the quality of life of patients in a highly sensitive moment.

**Conclusion:**

We conclude that personal aspects of the physician define their actions more than knowledge or organized structure. It is thus necessary that pediatric oncologists receive ethics and humanistic education.

## Introduction

The incidence of cancer in the pediatric age group has increased over the past 50 years (1). In 2021, an estimated 15,590 new cases of cancer will be diagnosed in children under 19 years of age in the United States of America (2). Currently, 75%–80% of children with cancer in developed countries will achieve remission (1). Those who do not enter remission will die of cancer or related complications. These patients should receive adequate palliative care to manage their symptoms and treat their needs until death.

Palliative care is not always provided correctly (3, 4), which decreases quality of life and leads to therapeutic futility. This can be explained by a lack of healthcare provider skills in addressing symptoms, infrequent use of systematic assessment tools, and uncertainty regarding the accuracy of patient-reported symptoms (5). The lack of ethical preparation for physicians has also been described (6, 7).

The teaching of virtue-based medical ethics does not figure significantly in university curricula. In contrast, it was reported in a study by Kotzee that, although the curriculum in the United Kingdom is intended to train virtuous physicians, most of the content of the core curriculum assumes the understanding that students must demonstrate professionalism and an understanding of patients' rights, consent, and capacity, among others, in their courses, and no particular attention is given to the development of virtues (8).

A considerable number of healthcare providers, including pediatric oncologists, are unclear about the definitions and application of end-of-life medical decisions, leading to the patient's needs being met late or at the wrong time. The status of end-of-life medical decisions generally depends on the category to which they belong: (i) maintaining or withdrawing life-sustaining treatments (non-treatment decisions), refusal of treatment, and limitation of therapeutic effort; (ii) intensified pain and symptom relief (such as palliative sedation); and (iii) provision, prescription, or administration of lethal drugs by a physician with the explicit intent to end the patient's life (euthanasia and medically assisted suicide) (9).

In the last two decades, there has been only modest improvement in the treatment of symptoms and suffering during the end of life for children dying of cancer (4). Pediatric oncologists are trained in the diagnosis, prognosis, evaluation, treatment, and follow-up of their patients. However, when confronted with ethical issues, they make medical decisions based on their own experiences rather than on precise knowledge of these issues. The factors that determine this experience usually include the physician's age, the number of years of practice, the number of patients treated in these circumstances, their beliefs, religion, ethical training, local legislation, cultural aspects, and their own values.

Moreover, these decisions are made not only at the end of life but throughout the process. Even when there is only a suspicion of cancer in the patient, the physician must make decisions that aim to protect the best interests of the child. Ethical aspects are also involved during this process, requiring greater participation of the patient and their parents in the doctor-patient relationship. Children with cancer experience changes in their perception of death as they become aware of their disease and its complications, which means that the pediatric oncologist must include in their decisions the patient's preferences, culture, education, beliefs, values, expectations of life, and their experience at the end of life in addition to their physical condition.

Physicians' lack of knowledge regarding end-of-life decisions leads to ethical dilemmas. Misinterpretation of these decisions can cause suffering or symptoms that diminish the quality of life of patients and lead to measures that do not meet the needs of the patient and family, leading to a lack of treatment of symptoms or therapeutic futility, which disrupts adequate management of the physical, psychological, social, and spiritual needs of the patient and their family. The actions and interventions the treating physician decide on change throughout the course of the disease, and the goals must be focused on the management and treatment of the needs of the child and family.

During the disease process—including the end of life—physicians and parents must take into account the possibility of short-and long-term mortality, the burden of treatment, family interests, and prognostic uncertainty as well as whether or not these are clearly related to the best interests of the child (10). The dynamics of decision-making during the disease process shape the therapeutic and care measures that will be provided to the patient. This implies that the factors involved in these decisions are weighted differently in each clinical situation. This variable-weighting may result in the child being subjected to intensive treatment and prolonged hospitalization (10).

The aim of this systematic review is to synthesize research findings regarding pediatric oncology end-of-life decision-making for children and adolescents with cancer using a comprehensive bioethical perspective. The study explores bioethics as an approach to guide the complexity of end-of-life decision-making for pediatric cancer patients. Our cross-functional approach identifies the state of the art of medical decision-making and is intended to improve the understanding of end-of-life bioethical deliberation regarding pediatric cancer patients. We report a systematic review that includes (a) classification of articles screened by the type of decision-making studied, (b) the value system at play in end-of-life decision-making, and (c) ethical discernment of major clinical practice dilemmas in oncology. In addition, we present the contributions of the bioethical approach from an integral perspective in different scenarios at these patients' end of life as well as a general outline of the processes that are carried out throughout the models and their application. The study concludes with a discussion of best practices in bioethical discernment and decision-making at the end of life.

## Materials and Methods

### Literature Review

#### Criteria for Considering Studies for This Review

##### Search Strategies

The search strategy was based on the modified approach PICO (Participants, Intervention, and Outcome) coupled with the principles of the Preferred Reporting Items for Systematic Review and Meta-Analyses (PRISMA) as a validation approach (11). We use both approaches to achieve more stringent conditions. The PIO strategy, which includes participant or problem, intervention or exposure, and outcomes, was used to systematically search a variety of different databases (PubMed, Embase, BIREME, EBM, and the Reviews and Philosophers Index) and requires more keywords to complete all the MeSH terms (Medical Subject Heading) to obtain an integrative dimension of the research question. We included all types of publications, except for case reports. The last search was conducted in September 2020. Articles in English were included in the analysis. One of the articles added by the researchers for discussion was published in Polish and was used after translation by collaborators of the researchers.

A search document was created using Medical Subject Heading (MeSH) terms and Boolean operators with the criteria previously described to obtain the articles. To analyze these results, they were grouped in a decision tree scheme for each database. This search strategy is described in **Table 1**.

**Table 1:** Search strategy for PUBMED.

**PARTICIPANTS**
**Children = 2701897**
**Adolescents = 2133220**
**Cancer = 4170135**
**End of life = 84194**
P
**Children OR adolescents AND cancer AND end of life = 1994**
(((((((("child"[MeSH Terms] OR "child"[All Fields]) OR "children"[All Fields]) OR "child s"[All Fields]) OR "children s"[All Fields]) OR "childrens"[All Fields]) OR "childs"[All Fields]) OR (((((("adolescences"[All Fields] OR "adolescency"[All Fields]) OR "adolescent"[MeSH Terms]) OR "adolescent"[All Fields]) OR "adolescence"[All Fields]) OR "adolescents"[All Fields]) OR "adolescent s"[All Fields])) AND ((((((((("cancer s"[All Fields] OR "cancerated"[All Fields]) OR "canceration"[All Fields]) OR "cancerization"[All Fields]) OR "cancerized"[All Fields]) OR "cancerous"[All Fields]) OR "neoplasms"[MeSH Terms]) OR "neoplasms"[All Fields]) OR "cancer"[All Fields]) OR "cancers"[All Fields])) AND ("end"[All Fields] AND ("life"[MeSH Terms] OR "life"[All Fields]))
**INTERVENTION**
**Medical decision making = 118568**
**Ethical discernment = 566**
**Ethical deliberation = 2099**
**Treatment refusal = 16075**
**Medical futility = 4053**
**Palliative sedation = 1339**
**Limitation therapeutic effort = 13012**
**Passive euthanasia = 6157**
**Medical decision making OR ethical discernment OR ethical deliberation = 120782**
((((("clinical decision-making"[MeSH Terms] OR ("clinical"[All Fields] AND "decision making"[All Fields])) OR "clinical decision making"[All Fields]) OR (("medical"[All Fields] AND "decision"[All Fields]) AND "making"[All Fields])) OR "medical decision making"[All Fields]) OR (((((((((("ethic s"[All Fields] OR "ethicality"[All Fields]) OR "ethically"[All Fields]) OR "ethics"[MeSH Terms]) OR "ethics"[All Fields]) OR "ethic"[All Fields]) OR "ethics"[MeSH Subheading]) OR "morals"[MeSH Terms]) OR "morals"[All Fields]) OR "ethical"[All Fields]) AND (((((("discern"[All Fields] OR "discernable"[All Fields]) OR "discerned"[All Fields]) OR "discernible"[All Fields]) OR "discerning"[All Fields]) OR "discernment"[All Fields]) OR "discerns"[All Fields]))) OR (((((((((("ethic s"[All Fields] OR "ethicality"[All Fields]) OR "ethically"[All Fields]) OR "ethics"[MeSH Terms]) OR "ethics"[All Fields]) OR "ethic"[All Fields]) OR "ethics"[MeSH Subheading]) OR "morals"[MeSH Terms]) OR "morals"[All Fields]) OR "ethical"[All Fields]) AND (((((("deliberate"[All Fields] OR "deliberated"[All Fields]) OR "deliberately"[All Fields]) OR "deliberates"[All Fields]) OR "deliberating"[All Fields]) OR "deliberation"[All Fields]) OR "deliberations"[All Fields]))
**Medical decision making OR ethical discernment OR ethical deliberation AND treatment refusal = 1440**
(((((("clinical decision-making"[MeSH Terms] OR ("clinical"[All Fields] AND "decision making"[All Fields])) OR "clinical decision making"[All Fields]) OR (("medical"[All Fields] AND "decision"[All Fields]) AND "making"[All Fields])) OR "medical decision making"[All Fields]) OR (((((((((("ethic s"[All Fields] OR "ethicality"[All Fields]) OR "ethically"[All Fields]) OR "ethics"[MeSH Terms]) OR "ethics"[All Fields]) OR "ethic"[All Fields]) OR "ethics"[MeSH Subheading]) OR "morals"[MeSH Terms]) OR "morals"[All Fields]) OR "ethical"[All Fields]) AND (((((("discern"[All Fields] OR "discernable"[All Fields]) OR "discerned"[All Fields]) OR "discernible"[All Fields]) OR "discerning"[All Fields]) OR "discernment"[All Fields]) OR "discerns"[All Fields]))) OR (((((((((("ethic s"[All Fields] OR "ethicality"[All Fields]) OR "ethically"[All Fields]) OR "ethics"[MeSH Terms]) OR "ethics"[All Fields]) OR "ethic"[All Fields]) OR "ethics"[MeSH Subheading]) OR "morals"[MeSH Terms]) OR "morals"[All Fields]) OR "ethical"[All Fields]) AND (((((("deliberate"[All Fields] OR "deliberated"[All Fields]) OR "deliberately"[All Fields]) OR "deliberates"[All Fields]) OR "deliberating"[All Fields]) OR "deliberation"[All Fields]) OR "deliberations"[All Fields]))) AND (("treatment refusal"[MeSH Terms] OR ("treatment"[All Fields] AND "refusal"[All Fields])) OR "treatment refusal"[All Fields])
**Medical decision making OR ethical discernment OR ethical deliberation AND medical futility = 1262**
(((((("clinical decision-making"[MeSH Terms] OR ("clinical"[All Fields] AND "decision making"[All Fields])) OR "clinical decision making"[All Fields]) OR (("medical"[All Fields] AND "decision"[All Fields]) AND "making"[All Fields])) OR "medical decision making"[All Fields]) OR (((((((((("ethic s"[All Fields] OR "ethicality"[All Fields]) OR "ethically"[All Fields]) OR "ethics"[MeSH Terms]) OR "ethics"[All Fields]) OR "ethic"[All Fields]) OR "ethics"[MeSH Subheading]) OR "morals"[MeSH Terms]) OR "morals"[All Fields]) OR "ethical"[All Fields]) AND (((((("discern"[All Fields] OR "discernable"[All Fields]) OR "discerned"[All Fields]) OR "discernible"[All Fields]) OR "discerning"[All Fields]) OR "discernment"[All Fields]) OR "discerns"[All Fields]))) OR (((((((((("ethic s"[All Fields] OR "ethicality"[All Fields]) OR "ethically"[All Fields]) OR "ethics"[MeSH Terms]) OR "ethics"[All Fields]) OR "ethic"[All Fields]) OR "ethics"[MeSH Subheading]) OR "morals"[MeSH Terms]) OR "morals"[All Fields]) OR "ethical"[All Fields]) AND (((((("deliberate"[All Fields] OR "deliberated"[All Fields]) OR "deliberately"[All Fields]) OR "deliberates"[All Fields]) OR "deliberating"[All Fields]) OR "deliberation"[All Fields]) OR "deliberations"[All Fields]))) AND (("medical futility"[MeSH Terms] OR ("medical"[All Fields] AND "futility"[All Fields])) OR "medical futility"[All Fields])
**Medical decision making OR ethical discernment OR ethical deliberation AND palliative sedation = 175**
(((((("clinical decision-making"[MeSH Terms] OR ("clinical"[All Fields] AND "decision making"[All Fields])) OR "clinical decision making"[All Fields]) OR (("medical"[All Fields] AND "decision"[All Fields]) AND "making"[All Fields])) OR "medical decision making"[All Fields]) OR (((((((((("ethic s"[All Fields] OR "ethicality"[All Fields]) OR "ethically"[All Fields]) OR "ethics"[MeSH Terms]) OR "ethics"[All Fields]) OR "ethic"[All Fields]) OR "ethics"[MeSH Subheading]) OR "morals"[MeSH Terms]) OR "morals"[All Fields]) OR "ethical"[All Fields]) AND (((((("discern"[All Fields] OR "discernable"[All Fields]) OR "discerned"[All Fields]) OR "discernible"[All Fields]) OR "discerning"[All Fields]) OR "discernment"[All Fields]) OR "discerns"[All Fields]))) OR (((((((((("ethic s"[All Fields] OR "ethicality"[All Fields]) OR "ethically"[All Fields]) OR "ethics"[MeSH Terms]) OR "ethics"[All Fields]) OR "ethic"[All Fields]) OR "ethics"[MeSH Subheading]) OR "morals"[MeSH Terms]) OR "morals"[All Fields]) OR "ethical"[All Fields]) AND (((((("deliberate"[All Fields] OR "deliberated"[All Fields]) OR "deliberately"[All Fields]) OR "deliberates"[All Fields]) OR "deliberating"[All Fields]) OR "deliberation"[All Fields]) OR "deliberations"[All Fields]))) AND ((("palliative"[All Fields] OR "palliatively"[All Fields]) OR "palliatives"[All Fields]) AND (((("sedate"[All Fields] OR "sedated"[All Fields]) OR "sedating"[All Fields]) OR "sedation"[All Fields]) OR "sedations"[All Fields]))
**Medical decision making OR ethical discernment OR ethical deliberation AND limitation therapeutic effort = 302**
(((((("clinical decision-making"[MeSH Terms] OR ("clinical"[All Fields] AND "decision making"[All Fields])) OR "clinical decision making"[All Fields]) OR (("medical"[All Fields] AND "decision"[All Fields]) AND "making"[All Fields])) OR "medical decision making"[All Fields]) OR (((((((((("ethic s"[All Fields] OR "ethicality"[All Fields]) OR "ethically"[All Fields]) OR "ethics"[MeSH Terms]) OR "ethics"[All Fields]) OR "ethic"[All Fields]) OR "ethics"[MeSH Subheading]) OR "morals"[MeSH Terms]) OR "morals"[All Fields]) OR "ethical"[All Fields]) AND (((((("discern"[All Fields] OR "discernable"[All Fields]) OR "discerned"[All Fields]) OR "discernible"[All Fields]) OR "discerning"[All Fields]) OR "discernment"[All Fields]) OR "discerns"[All Fields]))) OR (((((((((("ethic s"[All Fields] OR "ethicality"[All Fields]) OR "ethically"[All Fields]) OR "ethics"[MeSH Terms]) OR "ethics"[All Fields]) OR "ethic"[All Fields]) OR "ethics"[MeSH Subheading]) OR "morals"[MeSH Terms]) OR "morals"[All Fields]) OR "ethical"[All Fields]) AND (((((("deliberate"[All Fields] OR "deliberated"[All Fields]) OR "deliberately"[All Fields]) OR "deliberates"[All Fields]) OR "deliberating"[All Fields]) OR "deliberation"[All Fields]) OR "deliberations"[All Fields]))) AND (((((("limit"[All Fields] OR "limitation"[All Fields]) OR "limitations"[All Fields]) OR "limited"[All Fields]) OR "limiting"[All Fields]) OR "limits"[All Fields]) AND ((((("therapeutical"[All Fields] OR "therapeutically"[All Fields]) OR "therapeuticals"[All Fields]) OR "therapeutics"[MeSH Terms]) OR "therapeutics"[All Fields]) OR "therapeutic"[All Fields]) AND ("effort"[All Fields] OR "efforts"[All Fields]))
**Medical decision making OR ethical discernment OR ethical deliberation AND passive euthanasia = 1238**
(((((("clinical decision-making"[MeSH Terms] OR ("clinical"[All Fields] AND "decision making"[All Fields])) OR "clinical decision making"[All Fields]) OR (("medical"[All Fields] AND "decision"[All Fields]) AND "making"[All Fields])) OR "medical decision making"[All Fields]) OR (((((((((("ethic s"[All Fields] OR "ethicality"[All Fields]) OR "ethically"[All Fields]) OR "ethics"[MeSH Terms]) OR "ethics"[All Fields]) OR "ethic"[All Fields]) OR "ethics"[MeSH Subheading]) OR "morals"[MeSH Terms]) OR "morals"[All Fields]) OR "ethical"[All Fields]) AND (((((("discern"[All Fields] OR "discernable"[All Fields]) OR "discerned"[All Fields]) OR "discernible"[All Fields]) OR "discerning"[All Fields]) OR "discernment"[All Fields]) OR "discerns"[All Fields]))) OR (((((((((("ethic s"[All Fields] OR "ethicality"[All Fields]) OR "ethically"[All Fields]) OR "ethics"[MeSH Terms]) OR "ethics"[All Fields]) OR "ethic"[All Fields]) OR "ethics"[MeSH Subheading]) OR "morals"[MeSH Terms]) OR "morals"[All Fields]) OR "ethical"[All Fields]) AND (((((("deliberate"[All Fields] OR "deliberated"[All Fields]) OR "deliberately"[All Fields]) OR "deliberates"[All Fields]) OR "deliberating"[All Fields]) OR "deliberation"[All Fields]) OR "deliberations"[All Fields]))) AND ((("euthanasia, passive"[MeSH Terms] OR ("euthanasia"[All Fields] AND "passive"[All Fields])) OR "passive euthanasia"[All Fields]) OR ("passive"[All Fields] AND "euthanasia"[All Fields]))
**Treatment refusal OR medical futility OR palliative sedation OR limitation therapeutic effort OR passive euthanasia = 38567**
((((("treatment refusal"[MeSH Terms] OR ("treatment"[All Fields] AND "refusal"[All Fields])) OR "treatment refusal"[All Fields]) OR (("medical futility"[MeSH Terms] OR ("medical"[All Fields] AND "futility"[All Fields])) OR "medical futility"[All Fields])) OR ((("palliative"[All Fields] OR "palliatively"[All Fields]) OR "palliatives"[All Fields]) AND (((("sedate"[All Fields] OR "sedated"[All Fields]) OR "sedating"[All Fields]) OR "sedation"[All Fields]) OR "sedations"[All Fields]))) OR (((((("limit"[All Fields] OR "limitation"[All Fields]) OR "limitations"[All Fields]) OR "limited"[All Fields]) OR "limiting"[All Fields]) OR "limits"[All Fields]) AND ((((("therapeutical"[All Fields] OR "therapeutically"[All Fields]) OR "therapeuticals"[All Fields]) OR "therapeutics"[MeSH Terms]) OR "therapeutics"[All Fields]) OR "therapeutic"[All Fields]) AND ("effort"[All Fields] OR "efforts"[All Fields]))) OR ((("euthanasia, passive"[MeSH Terms] OR ("euthanasia"[All Fields] AND "passive"[All Fields])) OR "passive euthanasia"[All Fields]) OR ("passive"[All Fields] AND "euthanasia"[All Fields]))
I
**(Medical decision making OR ethical discernment OR ethical deliberation) AND (treatment refusal OR medical futility OR palliative sedation OR limitation therapeutic effort OR passive euthanasia) = 3725**
(((((("clinical decision-making"[MeSH Terms] OR ("clinical"[All Fields] AND "decision making"[All Fields])) OR "clinical decision making"[All Fields]) OR (("medical"[All Fields] AND "decision"[All Fields]) AND "making"[All Fields])) OR "medical decision making"[All Fields]) OR (((((((((("ethic s"[All Fields] OR "ethicality"[All Fields]) OR "ethically"[All Fields]) OR "ethics"[MeSH Terms]) OR "ethics"[All Fields]) OR "ethic"[All Fields]) OR "ethics"[MeSH Subheading]) OR "morals"[MeSH Terms]) OR "morals"[All Fields]) OR "ethical"[All Fields]) AND (((((("discern"[All Fields] OR "discernable"[All Fields]) OR "discerned"[All Fields]) OR "discernible"[All Fields]) OR "discerning"[All Fields]) OR "discernment"[All Fields]) OR "discerns"[All Fields]))) OR (((((((((("ethic s"[All Fields] OR "ethicality"[All Fields]) OR "ethically"[All Fields]) OR "ethics"[MeSH Terms]) OR "ethics"[All Fields]) OR "ethic"[All Fields]) OR "ethics"[MeSH Subheading]) OR "morals"[MeSH Terms]) OR "morals"[All Fields]) OR "ethical"[All Fields]) AND (((((("deliberate"[All Fields] OR "deliberated"[All Fields]) OR "deliberately"[All Fields]) OR "deliberates"[All Fields]) OR "deliberating"[All Fields]) OR "deliberation"[All Fields]) OR "deliberations"[All Fields]))) AND (((((("treatment refusal"[MeSH Terms] OR ("treatment"[All Fields] AND "refusal"[All Fields])) OR "treatment refusal"[All Fields]) OR (("medical futility"[MeSH Terms] OR ("medical"[All Fields] AND "futility"[All Fields])) OR "medical futility"[All Fields])) OR ((("palliative"[All Fields] OR "palliatively"[All Fields]) OR "palliatives"[All Fields]) AND (((("sedate"[All Fields] OR "sedated"[All Fields]) OR "sedating"[All Fields]) OR "sedation"[All Fields]) OR "sedations"[All Fields]))) OR (((((("limit"[All Fields] OR "limitation"[All Fields]) OR "limitations"[All Fields]) OR "limited"[All Fields]) OR "limiting"[All Fields]) OR "limits"[All Fields]) AND ((((("therapeutical"[All Fields] OR "therapeutically"[All Fields]) OR "therapeuticals"[All Fields]) OR "therapeutics"[MeSH Terms]) OR "therapeutics"[All Fields]) OR "therapeutic"[All Fields]) AND ("effort"[All Fields] OR "efforts"[All Fields]))) OR ((("euthanasia, passive"[MeSH Terms] OR ("euthanasia"[All Fields] AND "passive"[All Fields])) OR "passive euthanasia"[All Fields]) OR ("passive"[All Fields] AND "euthanasia"[All Fields])))
PI
**(Children OR adolescents AND cancer AND end of life) AND (Medical decision making OR ethical discernment OR ethical deliberation AND treatment refusal OR medical futility OR palliative sedation OR limitation therapeutic effort OR passive euthanasia) = 12**
((((((("clinical decision-making"[MeSH Terms] OR ("clinical"[All Fields] AND "decision making"[All Fields])) OR "clinical decision making"[All Fields]) OR (("medical"[All Fields] AND "decision"[All Fields]) AND "making"[All Fields])) OR "medical decision making"[All Fields]) OR (((((((((("ethic s"[All Fields] OR "ethicality"[All Fields]) OR "ethically"[All Fields]) OR "ethics"[MeSH Terms]) OR "ethics"[All Fields]) OR "ethic"[All Fields]) OR "ethics"[MeSH Subheading]) OR "morals"[MeSH Terms]) OR "morals"[All Fields]) OR "ethical"[All Fields]) AND (((((("discern"[All Fields] OR "discernable"[All Fields]) OR "discerned"[All Fields]) OR "discernible"[All Fields]) OR "discerning"[All Fields]) OR "discernment"[All Fields]) OR "discerns"[All Fields]))) OR (((((((((("ethic s"[All Fields] OR "ethicality"[All Fields]) OR "ethically"[All Fields]) OR "ethics"[MeSH Terms]) OR "ethics"[All Fields]) OR "ethic"[All Fields]) OR "ethics"[MeSH Subheading]) OR "morals"[MeSH Terms]) OR "morals"[All Fields]) OR "ethical"[All Fields]) AND (((((("deliberate"[All Fields] OR "deliberated"[All Fields]) OR "deliberately"[All Fields]) OR "deliberates"[All Fields]) OR "deliberating"[All Fields]) OR "deliberation"[All Fields]) OR "deliberations"[All Fields]))) AND (((((("treatment refusal"[MeSH Terms] OR ("treatment"[All Fields] AND "refusal"[All Fields])) OR "treatment refusal"[All Fields]) OR (("medical futility"[MeSH Terms] OR ("medical"[All Fields] AND "futility"[All Fields])) OR "medical futility"[All Fields])) OR ((("palliative"[All Fields] OR "palliatively"[All Fields]) OR "palliatives"[All Fields]) AND (((("sedate"[All Fields] OR "sedated"[All Fields]) OR "sedating"[All Fields]) OR "sedation"[All Fields]) OR "sedations"[All Fields]))) OR (((((("limit"[All Fields] OR "limitation"[All Fields]) OR "limitations"[All Fields]) OR "limited"[All Fields]) OR "limiting"[All Fields]) OR "limits"[All Fields]) AND ((((("therapeutical"[All Fields] OR "therapeutically"[All Fields]) OR "therapeuticals"[All Fields]) OR "therapeutics"[MeSH Terms]) OR "therapeutics"[All Fields]) OR "therapeutic"[All Fields]) AND ("effort"[All Fields] OR "efforts"[All Fields]))) OR ((("euthanasia, passive"[MeSH Terms] OR ("euthanasia"[All Fields] AND "passive"[All Fields])) OR "passive euthanasia"[All Fields]) OR ("passive"[All Fields] AND "euthanasia"[All Fields])))) AND ((((((((("child"[MeSH Terms] OR "child"[All Fields]) OR "children"[All Fields]) OR "child s"[All Fields]) OR "children s"[All Fields]) OR "childrens"[All Fields]) OR "childs"[All Fields]) OR (((((("adolescences"[All Fields] OR "adolescency"[All Fields]) OR "adolescent"[MeSH Terms]) OR "adolescent"[All Fields]) OR "adolescence"[All Fields]) OR "adolescents"[All Fields]) OR "adolescent s"[All Fields])) AND ((((((((("cancer s"[All Fields] OR "cancerated"[All Fields]) OR "canceration"[All Fields]) OR "cancerization"[All Fields]) OR "cancerized"[All Fields]) OR "cancerous"[All Fields]) OR "neoplasms"[MeSH Terms]) OR "neoplasms"[All Fields]) OR "cancer"[All Fields]) OR "cancers"[All Fields])) AND ("end"[All Fields] AND ("life"[MeSH Terms] OR "life"[All Fields])))
**OUTCOME**
**Quality of life = 404402**
**Patient satisfaction = 133791**
**Family satisfaction = 30119**
**Hospitalization time = 941968**
**Comorbidity = 249172**
**Dignity = 394546**
**Dying dignity = 1385**
O
**Quality of life OR patient satisfaction OR Family satisfaction OR hospitalization time OR comorbidity OR dignity OR dying dignity = 1982901**
((((((("quality of life"[MeSH Terms] OR ("quality"[All Fields] AND "life"[All Fields])) OR "quality of life"[All Fields]) OR (("patient satisfaction"[MeSH Terms] OR ("patient"[All Fields] AND "satisfaction"[All Fields])) OR "patient satisfaction"[All Fields])) OR ((((((((((("familialities"[All Fields] OR "familiality"[All Fields]) OR "familially"[All Fields]) OR "familials"[All Fields]) OR "familie"[All Fields]) OR "family"[MeSH Terms]) OR "family"[All Fields]) OR "familial"[All Fields]) OR "families"[All Fields]) OR "family s"[All Fields]) OR "familys"[All Fields]) AND (((("personal satisfaction"[MeSH Terms] OR ("personal"[All Fields] AND "satisfaction"[All Fields])) OR "personal satisfaction"[All Fields]) OR "satisfaction"[All Fields]) OR "satisfactions"[All Fields]))) OR ((((((((((((((("hospital s"[All Fields] OR "hospitalisation"[All Fields]) OR "hospitalization"[MeSH Terms]) OR "hospitalization"[All Fields]) OR "hospitalised"[All Fields]) OR "hospitalising"[All Fields]) OR "hospitality"[All Fields]) OR "hospitalisations"[All Fields]) OR "hospitalizations"[All Fields]) OR "hospitalize"[All Fields]) OR "hospitalized"[All Fields]) OR "hospitalizing"[All Fields]) OR "hospitals"[MeSH Terms]) OR "hospitals"[All Fields]) OR "hospital"[All Fields]) AND ("time"[MeSH Terms] OR "time"[All Fields]))) OR (((("comorbid"[All Fields] OR "comorbidity"[MeSH Terms]) OR "comorbidity"[All Fields]) OR "comorbidities"[All Fields]) OR "comorbids"[All Fields])) OR ((("dignities"[All Fields] OR "respect"[MeSH Terms]) OR "respect"[All Fields]) OR "dignity"[All Fields])) OR ("dying"[All Fields] AND ((("dignities"[All Fields] OR "respect"[MeSH Terms]) OR "respect"[All Fields]) OR "dignity"[All Fields]))
IO
**(Medical decision making OR ethical discernment OR ethical deliberation) AND (treatment refusal OR medical futility OR palliative sedation OR limitation therapeutic effort OR passive euthanasia) AND (Quality of life OR patient satisfaction OR Family satisfaction OR hospitalization time OR comorbidity OR dignity OR dying dignity) = 1023**
((((((("clinical decision-making"[MeSH Terms] OR ("clinical"[All Fields] AND "decision making"[All Fields])) OR "clinical decision making"[All Fields]) OR (("medical"[All Fields] AND "decision"[All Fields]) AND "making"[All Fields])) OR "medical decision making"[All Fields]) OR (((((((((("ethic s"[All Fields] OR "ethicality"[All Fields]) OR "ethically"[All Fields]) OR "ethics"[MeSH Terms]) OR "ethics"[All Fields]) OR "ethic"[All Fields]) OR "ethics"[MeSH Subheading]) OR "morals"[MeSH Terms]) OR "morals"[All Fields]) OR "ethical"[All Fields]) AND (((((("discern"[All Fields] OR "discernable"[All Fields]) OR "discerned"[All Fields]) OR "discernible"[All Fields]) OR "discerning"[All Fields]) OR "discernment"[All Fields]) OR "discerns"[All Fields]))) OR (((((((((("ethic s"[All Fields] OR "ethicality"[All Fields]) OR "ethically"[All Fields]) OR "ethics"[MeSH Terms]) OR "ethics"[All Fields]) OR "ethic"[All Fields]) OR "ethics"[MeSH Subheading]) OR "morals"[MeSH Terms]) OR "morals"[All Fields]) OR "ethical"[All Fields]) AND (((((("deliberate"[All Fields] OR "deliberated"[All Fields]) OR "deliberately"[All Fields]) OR "deliberates"[All Fields]) OR "deliberating"[All Fields]) OR "deliberation"[All Fields]) OR "deliberations"[All Fields]))) AND (((((("treatment refusal"[MeSH Terms] OR ("treatment"[All Fields] AND "refusal"[All Fields])) OR "treatment refusal"[All Fields]) OR (("medical futility"[MeSH Terms] OR ("medical"[All Fields] AND "futility"[All Fields])) OR "medical futility"[All Fields])) OR ((("palliative"[All Fields] OR "palliatively"[All Fields]) OR "palliatives"[All Fields]) AND (((("sedate"[All Fields] OR "sedated"[All Fields]) OR "sedating"[All Fields]) OR "sedation"[All Fields]) OR "sedations"[All Fields]))) OR (((((("limit"[All Fields] OR "limitation"[All Fields]) OR "limitations"[All Fields]) OR "limited"[All Fields]) OR "limiting"[All Fields]) OR "limits"[All Fields]) AND ((((("therapeutical"[All Fields] OR "therapeutically"[All Fields]) OR "therapeuticals"[All Fields]) OR "therapeutics"[MeSH Terms]) OR "therapeutics"[All Fields]) OR "therapeutic"[All Fields]) AND ("effort"[All Fields] OR "efforts"[All Fields]))) OR ((("euthanasia, passive"[MeSH Terms] OR ("euthanasia"[All Fields] AND "passive"[All Fields])) OR "passive euthanasia"[All Fields]) OR ("passive"[All Fields] AND "euthanasia"[All Fields])))) AND (((((((("quality of life"[MeSH Terms] OR ("quality"[All Fields] AND "life"[All Fields])) OR "quality of life"[All Fields]) OR (("patient satisfaction"[MeSH Terms] OR ("patient"[All Fields] AND "satisfaction"[All Fields])) OR "patient satisfaction"[All Fields])) OR ((((((((((("familialities"[All Fields] OR "familiality"[All Fields]) OR "familially"[All Fields]) OR "familials"[All Fields]) OR "familie"[All Fields]) OR "family"[MeSH Terms]) OR "family"[All Fields]) OR "familial"[All Fields]) OR "families"[All Fields]) OR "family s"[All Fields]) OR "familys"[All Fields]) AND (((("personal satisfaction"[MeSH Terms] OR ("personal"[All Fields] AND "satisfaction"[All Fields])) OR "personal satisfaction"[All Fields]) OR "satisfaction"[All Fields]) OR "satisfactions"[All Fields]))) OR ((((((((((((((("hospital s"[All Fields] OR "hospitalisation"[All Fields]) OR "hospitalization"[MeSH Terms]) OR "hospitalization"[All Fields]) OR "hospitalised"[All Fields]) OR "hospitalising"[All Fields]) OR "hospitality"[All Fields]) OR "hospitalisations"[All Fields]) OR "hospitalizations"[All Fields]) OR "hospitalize"[All Fields]) OR "hospitalized"[All Fields]) OR "hospitalizing"[All Fields]) OR "hospitals"[MeSH Terms]) OR "hospitals"[All Fields]) OR "hospital"[All Fields]) AND ("time"[MeSH Terms] OR "time"[All Fields]))) OR (((("comorbid"[All Fields] OR "comorbidity"[MeSH Terms]) OR "comorbidity"[All Fields]) OR "comorbidities"[All Fields]) OR "comorbids"[All Fields])) OR ((("dignities"[All Fields] OR "respect"[MeSH Terms]) OR "respect"[All Fields]) OR "dignity"[All Fields])) OR ("dying"[All Fields] AND ((("dignities"[All Fields] OR "respect"[MeSH Terms]) OR "respect"[All Fields]) OR "dignity"[All Fields])))
PIO
**(Children OR adolescents AND cancer AND end of life ) AND (Medical decision making OR ethical discernment OR ethical deliberation) AND (treatment refusal OR medical futility OR palliative sedation OR limitation therapeutic effort OR passive euthanasia) AND Quality of life OR patient satisfaction OR Family satisfaction OR hospitalization time OR comorbidity OR dignity OR dying dignity = 6**
(((((((((("child"[MeSH Terms] OR "child"[All Fields]) OR "children"[All Fields]) OR "child s"[All Fields]) OR "children s"[All Fields]) OR "childrens"[All Fields]) OR "childs"[All Fields]) OR (((((("adolescences"[All Fields] OR "adolescency"[All Fields]) OR "adolescent"[MeSH Terms]) OR "adolescent"[All Fields]) OR "adolescence"[All Fields]) OR "adolescents"[All Fields]) OR "adolescent s"[All Fields])) AND ((((((((("cancer s"[All Fields] OR "cancerated"[All Fields]) OR "canceration"[All Fields]) OR "cancerization"[All Fields]) OR "cancerized"[All Fields]) OR "cancerous"[All Fields]) OR "neoplasms"[MeSH Terms]) OR "neoplasms"[All Fields]) OR "cancer"[All Fields]) OR "cancers"[All Fields])) AND ("end"[All Fields] AND ("life"[MeSH Terms] OR "life"[All Fields]))) AND ((((((("clinical decision-making"[MeSH Terms] OR ("clinical"[All Fields] AND "decision making"[All Fields])) OR "clinical decision making"[All Fields]) OR (("medical"[All Fields] AND "decision"[All Fields]) AND "making"[All Fields])) OR "medical decision making"[All Fields]) OR (((((((((("ethic s"[All Fields] OR "ethicality"[All Fields]) OR "ethically"[All Fields]) OR "ethics"[MeSH Terms]) OR "ethics"[All Fields]) OR "ethic"[All Fields]) OR "ethics"[MeSH Subheading]) OR "morals"[MeSH Terms]) OR "morals"[All Fields]) OR "ethical"[All Fields]) AND (((((("discern"[All Fields] OR "discernable"[All Fields]) OR "discerned"[All Fields]) OR "discernible"[All Fields]) OR "discerning"[All Fields]) OR "discernment"[All Fields]) OR "discerns"[All Fields]))) OR (((((((((("ethic s"[All Fields] OR "ethicality"[All Fields]) OR "ethically"[All Fields]) OR "ethics"[MeSH Terms]) OR "ethics"[All Fields]) OR "ethic"[All Fields]) OR "ethics"[MeSH Subheading]) OR "morals"[MeSH Terms]) OR "morals"[All Fields]) OR "ethical"[All Fields]) AND (((((("deliberate"[All Fields] OR "deliberated"[All Fields]) OR "deliberately"[All Fields]) OR "deliberates"[All Fields]) OR "deliberating"[All Fields]) OR "deliberation"[All Fields]) OR "deliberations"[All Fields]))) AND (((((("treatment refusal"[MeSH Terms] OR ("treatment"[All Fields] AND "refusal"[All Fields])) OR "treatment refusal"[All Fields]) OR (("medical futility"[MeSH Terms] OR ("medical"[All Fields] AND "futility"[All Fields])) OR "medical futility"[All Fields])) OR ((("palliative"[All Fields] OR "palliatively"[All Fields]) OR "palliatives"[All Fields]) AND (((("sedate"[All Fields] OR "sedated"[All Fields]) OR "sedating"[All Fields]) OR "sedation"[All Fields]) OR "sedations"[All Fields]))) OR (((((("limit"[All Fields] OR "limitation"[All Fields]) OR "limitations"[All Fields]) OR "limited"[All Fields]) OR "limiting"[All Fields]) OR "limits"[All Fields]) AND ((((("therapeutical"[All Fields] OR "therapeutically"[All Fields]) OR "therapeuticals"[All Fields]) OR "therapeutics"[MeSH Terms]) OR "therapeutics"[All Fields]) OR "therapeutic"[All Fields]) AND ("effort"[All Fields] OR "efforts"[All Fields]))) OR ((("euthanasia, passive"[MeSH Terms] OR ("euthanasia"[All Fields] AND "passive"[All Fields])) OR "passive euthanasia"[All Fields]) OR ("passive"[All Fields] AND "euthanasia"[All Fields]))))) AND (((((((("quality of life"[MeSH Terms] OR ("quality"[All Fields] AND "life"[All Fields])) OR "quality of life"[All Fields]) OR (("patient satisfaction"[MeSH Terms] OR ("patient"[All Fields] AND "satisfaction"[All Fields])) OR "patient satisfaction"[All Fields])) OR ((((((((((("familialities"[All Fields] OR "familiality"[All Fields]) OR "familially"[All Fields]) OR "familials"[All Fields]) OR "familie"[All Fields]) OR "family"[MeSH Terms]) OR "family"[All Fields]) OR "familial"[All Fields]) OR "families"[All Fields]) OR "family s"[All Fields]) OR "familys"[All Fields]) AND (((("personal satisfaction"[MeSH Terms] OR ("personal"[All Fields] AND "satisfaction"[All Fields])) OR "personal satisfaction"[All Fields]) OR "satisfaction"[All Fields]) OR "satisfactions"[All Fields]))) OR ((((((((((((((("hospital s"[All Fields] OR "hospitalisation"[All Fields]) OR "hospitalization"[MeSH Terms]) OR "hospitalization"[All Fields]) OR "hospitalised"[All Fields]) OR "hospitalising"[All Fields]) OR "hospitality"[All Fields]) OR "hospitalisations"[All Fields]) OR "hospitalizations"[All Fields]) OR "hospitalize"[All Fields]) OR "hospitalized"[All Fields]) OR "hospitalizing"[All Fields]) OR "hospitals"[MeSH Terms]) OR "hospitals"[All Fields]) OR "hospital"[All Fields]) AND ("time"[MeSH Terms] OR "time"[All Fields]))) OR (((("comorbid"[All Fields] OR "comorbidity"[MeSH Terms]) OR "comorbidity"[All Fields]) OR "comorbidities"[All Fields]) OR "comorbids"[All Fields])) OR ((("dignities"[All Fields] OR "respect"[MeSH Terms]) OR "respect"[All Fields]) OR "dignity"[All Fields])) OR ("dying"[All Fields] AND ((("dignities"[All Fields] OR "respect"[MeSH Terms]) OR "respect"[All Fields]) OR "dignity"[All Fields])))

This table shows the construction of the research question. PIO strategy is described with the inclusion of: Participants. Children and adolescents, from 0 to 18 years old, with a cancer diagnostic at the End-of-life. Intervention. Taking account Ethical deliberation or discernment about the medical decisions at End-of-Life defined for the study (treatment refusal, medical futility, palliative sedation, limitation of therapeutic effort or passive euthanasia). Outcome. To evaluate consequences of the intervention we included quality of life, patient and family satisfaction, hospitalization time, comorbidity, dignity and dying dignity.

Because of our interest in circumstances, factors, values, and principles that may influence medical decision-making at the end of life in children or adolescents with cancer as well as how these decisions impact the quality of life of these patients and their families, we included articles that met the criteria outlined below.

### Criteria for Publication Inclusion

1. The study was conducted with children or adolescents, although we also included studies conducted in adolescents and young adults that analyzed decision-making and its consequences.
2. The authors of the study included an ethical framework relating the physician's profile, including values, medical and ethical training, experience, and other personal circumstances that influence end-of-life medical decision-making for children and adolescents with cancer.
3. The authors analyzed the physical, psychological, and social repercussions of end-of-life decision-making for children and adolescents with cancer.
4. The study included an instrument that explored the factors that determine medical decision-making at the end of life or the consequences of this decision-making for the patient or for the physician making the decision.
5. The study analyzed the factors that influence medical decision-making at the end of life for children and adolescents with cancer.

### Selection Criteria

The PIO (Participants, Intervention, and Outcome) (12) strategy criteria for study selection were as follows: type of participants, children or adolescents (from birth to 18 years of age) diagnosed with any type of cancer who were followed at the end of life; and type of intervention, including medical decisions, ethical discernment, and ethical deliberation. As medical decisions at the end of life, we included refusal of treatment, therapeutic futility, palliative sedation, limitation of therapeutic effort, and passive euthanasia. To evaluate the outcomes after decision-making, the following were included in the search: quality of life, patient and family satisfaction, length of hospitalization, comorbidity, dignity, and dignified death.

The qualitative criteria used the data fields predefined by the two principal investigators as proposed by Monroy et al., and adjusted to our aims (13) (**Table 2**. Criteria used to appraise study quality).

**Table 2:** Criteria used to appraise study quality.

	Quality criterion	Armstrong	Cicero-Oneto	Coyne	Delany	Hilden	Lotto	Sisk
1	Inclusion of children and adolescents, or adolescents/young adults	Y	Y	Y	Y	Y	Y	Y
2	Medical decision making at end of life	Y	Y	Y	Y	Y	Y	Y
3	Clear research questions and objectives	Y	Y	Y	Y	Y	Y	Y
4	Ethical discernment or ethical deliberation	Y	Y	N	Y	N	Y	Y
5	Method description in detail	Y	Y	Y	Y	Y	Y	Y
6	Outcome of patients after medical decision	N	Y	Y	Y	N	N	Y
7	Measure of satisfaction of patient or family	Y	Y	N	Y	Y	N	N
8	Medical values and factors that influence medical decisions at end of life addressed	Y	Y	Y	Y	Y	Y	Y
	Grade	87.5%	100%	75%	100%	75%	75%	87.5%

We excluded articles that discussed end-of-life medical decision-making for patients with diseases other than cancer.

### Eligibility Criteria

After the initial search, a first selection was made based on the title of the article, eliminating those with a topic irrelevant to our analysis. The second round of selection was conducted after reading the abstract. Finally, we obtained full-text articles that met the criteria for the analysis and discussion of the present systematic review.

To classify the quality criteria of the selected articles, we considered the country, the site (medical facility or hospital) where the study was carried out, the characteristics of the sample or population, the design of the study, the medical decision being analyzed, the factors that influenced the decision, the evolution or outcome, the application of an evaluation instrument, and the ethical dilemma being studied (**Table 3**. Classification of articles included in the systematic review).

**Table 3:** Classification of articles included in the systematic review with appraise study quality.

First author, year of publication	Site of study	Source or population	Main characteristics of source or population	Design	Medical decision	Factors influencing medical decisions	Outcome	Instrument or tool	Quality	Ethical dilemma
(14)	Department of Anesthesiology and Pain Medicine, University of Washington, USA	Two documents about a procedure of dispute resolution related to futility and non-beneficial interventions	Boston Children’s Hospital Futility Policy and Texas Advance Directives Act	Cross-sectional and descriptive	Medical futility, non-beneficial interventions	Clinicians avoid ethical, legal and clinical issues due their own concepts of values, emotions and knowledge about futility	Construction of a conceptual cohesive framework for discussion about futility	Design of an algorithm to help ease the discomfort of dealing with non-beneficial interventions	87.5%	Benefit of medical interventions
(15)	Hospital Infantil de México Federico Gómez, Mexico City, Mexico	13 pediatric oncologist, 13 parents or primary caregivers and 6 adolescents with incurable cancer	13 pediatric oncologist, 13 parents or primary caregivers and 6 adolescents with incurable cancer treated in three tertiary hospitals in Mexico City	Qualitative study based in semi-structured interviews	Medical futility	Training in palliative care, uncertain of disease prognosis, legal normative of ethical principles, prevalence of medical paternalism	Better understand of decision-making process and palliative care	Semi-structured face-to-face interview	100%	Withhold or withdraw treatment at end of life in adolescents with cancer
(16)	School of Nursing & Midwifery, Trinity College Dublin, Dublin, Ireland	Systematic review	3290 records identified, 2676 papers screened, 1 eligible full-text article, 0 studies identified	Systematic review of controlled and randomized studies, related to shared decision-making in children with cancer between 4 and 18 years	Shared decision-making	Lack of training of health professionals to develop communication skills	It is not clear which factors influence shared decision-making approach for children with cancer	None	75%	Shared decision-making
(17)	Children´s Bioethics Centre, Royal Children’s Hospital, Melbourne, Australia	18 health professionals	5 medical doctors with an average of 21 years of experience, 9 nurses with18 years of experience; 2 educational therapists, 1 chaplain and a social worker	Qualitative methodology based on the social theory of symbolic interactionism	Medical decisions at end of life	Ethical complexity, lack of availability of parental resources to defining life support at end of life	Usefulness of a handbook and web-based resource (Caring Decisions)	A handbook and an interview guide	100%	Withhold or withdraw advanced life support at end of life
(18)	Department of Pediatric Hematology/Oncology, Children’s Hospitals and Clinics-St Paul, St Paul, Minnesota, USA	228 pediatric oncologists, members of American Society of Clinical Oncology (ASCO)	All participants answered a 118 questions survey about end-of-life care in children with cancer	Logistic regression analysis to recognize predictors of attitudes and practices to face end-of-life	Palliative and end-of-life care	Sociodemographic predictors, age, sex, religious affiliation, importance of religious beliefs, recent death of a relative, specialty, type of practice, amount of time spent in patient care	Communication deficiencies between medical staff and parents, especially associated with decisions at end-of-life	Survey of 118 items, divided into 8 modules	75%	Withhold or withdraw of treatment at end-of-life
(19)	Department of Developmental Psychology and Socialization, University of Padova, Padova, Italy	202 under graduated students of the University of Padova	162 females, 40 males, between 18 to 29 years	Interpretation of 4 scenarios, related to the terms “alive” or “dead”, permanent vegetative state and minimally conscious state	Advanced planing directive	Perception of life status and principles of sanctity of life and free choice	A predictable correlation between people’s moral principles and the acceptance of withdrawing treatment in patient who request it	A 0 to 100 points scale to assess patient wishes	75%	Life support treatment in patients with brain damage
(20)	Department of Oncology, St Jude Children´s Research Hospital, Memphis, TN, USA	None	None	Ethical analysis	Medical decisions including treatment refusal, abandonment and end-of-life decisions	Bioethical principles, psychological issues and needs, Defective communication	It highlights the challenges of facing the particular needs of this age group	None	87.5%	Medical decisions

For the purposes of this study, end-of-life care, the decision-making process, the ethical values that were considered or deliberated, epistemic values, and ethical arguments with which these decisions were approached were also considered (**Table 4**. Axiological analysis of the articles of the systematic review).

**Table 4:** Axiological analysis of the articles of the systematic review.

Author, year of publication	End of life care	Decision making (Who, when, how)	Ethical values	Epistemic values	Ethical argumentation
Armstrong (14)	Avoid medical futility	Families and health professionals when risk-benefit relationship is high	Human dignity, autonomy, justive	To create a better definition of medical futility	Principialist bioethics, utilitarianism
Cicero (15)	Avoid medical futility	Pediatric oncologists and parents, at end of life, inducing medical futility in patients	Responsibility, mature minor doctrine	Encourage participation of adolescents with cancer in decision making at end-of-life, to avoid futile treatments	Principialist bioethics
Coyne (16)	All along disease care	Shared decision making between health professionals, parents and children with cancer throughout the treatment	Autonomy, best interests of children	Increase participation of children with cancer in decision-making during treatment, implement controlled clinical studies that support involvement of children in decision-making	Best interests of children
Delany (17)	Withdraw or withhold life support treatments	Shared decisions between health professionals and parents of critically illness children	Medical responsibility, honesty	Improve communication between parents and health professionals, using a textbook that help in decision-making	Medical responsibility
Lotto (19)	Treatment refusal, withdraw/withhold medical treatment at end-of-life	Medical doctors decide to withdraw/withhold treatment in patients with altered states of consciousness, based on patient free choice or sanctity of life	Autonomy, human dignity, human rights	Demonstrate a predictable correlation between the physicians’ moral principles and the agreement to withdraw life support treatment in patients who request it	Moral and ethical principles
Hilden (18)	Palliative care, end-of-life, euthanasia, and medically assisted suicide	Pediatric oncologists make decisions at end-of-life with poor knowledge about palliative care, medical ethics, euthanasia, and other medical decisions	Beneficence, dignity, human rights	Improve ethical training in pediatric oncologists for the better decision making at end-of-life	Medical ethics, responsibility ethics
Sisk (20)	Treatment refusal, abandonment, and adherence to treatment	Shared decision-making between parents and medical doctors, along all disease process	Justice, mature minor doctrine	Increase access to care during illness in adolescents and young adult patients	Principialist bioethics

### Synthesis: Qualitative Analysis

For the synthesis we imported the articles in the Atlas.ti v.9^®^ software and coded the research findings. We created code groups according to the following themes: medical decision, influencing factors, evolution, or outcome, and end of life care, decision-making process, ethical values considered or deliberated, epistemic values and ethical arguments. We defined networks to explore and develop a deeper synthesis to relate the information obtained graphically, observe the complexity of the phenomena, and clarify the relationship, co-occurrences, as proposed by Sueiras (7). Colors were used for each group of codes and the line that relate one code to another establishes the type of relationship between them. The bioethical analysis searched networks that could link decision-making to social, ethical, and professional values (**Table 5**. Atlas.ti v.9^®^ code groups).

**Table 5:** Atlas.ti^®^ code groups.

**ATLAS.ti Report Bioethical discernment at the End-Of-Life in children with cancer Group of codes** Created by Luis Juarez on 12 sep 2021
**Clinical determinants at End-Of-Life for Decision-Making 4 Codes: • Be alive • Consciousness • End-Of-Life care quality • Right to die**
** Decision-Making Clinical guides 12 Codes: • 4 steps of principled negotiation • Advanced care planning, directives • Boston Children's Hospital policy • Caring decision handbook • Children's rights United Nations • Directives of End-Of-Life care • Disputed Intervention, Texas Advance Directives Act (TADA) • Howard's model • Support material for decision making • Symbolic interactionism • Texas Advance Directives Act • Texas Advance Directives Act, protection to clinicians**
**Decisions 6 Codes: • Euthanasia Comment:** Intentionally terminating life by another person that the person concerned • **Futility Comment:** The unfortunate situation in which continued therapy will not benefit the patient and, therefore, ought not be used • **Limitation of therapeutic effort** **Comment:** Withdraw of medical treatments to avoid non beneficial support • **Palliative care** **Comment:** Palliative care in children, adolescents, and young adults with cancer has led to better outcomes for patients and their families, improved quality of life, and relief of suffering • **Palliative sedation** **Comment:** The use of sedative medications to relieve intractable and refractory distress by a reduction in patient consciousness • **Treatment refusal** **Comment:** Decision to avoid or not accept recommended elective treatment.
**Factors of Patients Decision-Making 6 Codes: • Adolescent and Young Adult (AYA) group cancer facts • AYA preferences, values, beliefs • Children decision making • Children involvement importance • Children's preferences • Patient values**
**Medical barriers 11 Codes: • Anxiety of Pediatric Oncologist • AYA barriers • Bad news • Communication skills of Pediatric Oncologist • Complexity of sharing information • Confusion in decision • Discrepancies of treatment • Obstacles to good care of dying children • Pediatric oncology security • Power relationship • Predictors of attitudes and practices**
**Medical factors to Decision-Making 63 Codes: • Anxiety of Pediatric Oncologist • Beliefs, emotions medics • Best interest standard • Biological decisions • Clinical judgement • Communication • Contexting decision • Decision making** **Comment:** A good decision is a logical decision -one based on the uncertainties, values, and preferences of the decision maker-. A good outcome is one that is profitable or otherwise highly valued. In short, a good outcome is one that we wish would happen.The distinction between decision and outcome is still not clear for most people • **Develop ethical understanding • Discussion about patient's values, prognosis, options and wishes • Dishonesty • Ethical deliberation • Ethical issues • Ethical training • Factors influence medical decision End-Of-Life • Failure feelings of Pediatric Oncologist • Free choice principle • health care providers knowledge • Honesty Comment:** Attribute of disclose accurately the extent of knowledge and ignorance •** Hospice service • Howard's model • Hubris absent • Human dignity • Interview structure to decision-making • Legality • Mature minor doctrine • Medical attitudes at End-Of-Life • Medical belief • Medical decision-making capacity • Medical deontology • Medical experience • Medical perception • Medical preconceptions • Medical satisfaction • Medical values • Medical-legally discussion • Medical-patient relationship • Moment to give bad news • Moment to give support material • Moral principles • Multidisciplinary consultation • Non-ethical standard • Oncologist time of experience • Paternalism • Philosophical aspects of End-Of-Life • Predictors of attitudes and practices • Prognostic decision • Prolonged physiologic life • Quality of information • Recognizing individualism • Sanctity of life • Satisfaction of Pediatric Oncologist about End-Of-Life care • Shared decision-making, age • Shared decision making (SDM) • Skills to manage symptoms • Social reality, medical decision • Training in palliative care • Understanding of decision • Unrealistic expectation • Who makes decisions • Withhold, withdraw life support • Work's place of Pediatric Oncologist • Wrong decision**
**Parents, patients’ factors for Decision-Making 26 Codes: • Beliefs, emotions parents • Cultural relation with decision making • Culture, decision making • Death, place, and time • Decision making involvement • Decisional quality • Disagreement patient-parent • Discussion with shared language • Empower parents • Ethics of families • Ethnic influences on decisions • Family decision making • Family influences about End-Of-Life decision • Health professionals behavioral • Information comprehension• Institution confidence • Interview structure to decision-making• Language • Outcome of shared decision-making • Parental values • Shared decision-making, age • Shared decision-making (SDM) • Understanding of decision • Unrealistic expectation of cure • Who makes decisions • Withhold, withdraw life support**
**Principles 4 Codes: • Beneficence Comment:** The act of contribution to the welfare of person or humanity • **Justice Comment:** Appropriate fair, equitable treatment in light of what is due or owed to persons • **Non maleficence Comment:** The obligation not to inflict harm on others • **Respect for autonomy Comment:** Individual decision making in health care, self-rule free from controlled interference
**Virtues 7 Codes: • Compassion Comment:** The disposition to comprehend, assess and weigh the uniqueness of patient´s predicament of illness • **Empathy • Honesty Comment:** Attribute of disclose accurately the extent of knowledge and ignorance • **Integrity Comment:** The person can integrate all the virtues into a whole and can prudentially judge to reach a decision to act • **Phronesis Comment:** The capacity of moral insight, the capacity to discern what moral choice or course of action is most conducive to the good • **Responsibility • Veracity Comment:** The capacity of tell the whole truth
**Codes without group 6 Codes: • Advance directive • Clinical trials participation • Duration, cost of medical futile • Futile mediation • Futility definition • Futility, treatment goals**

Report of all the groups of codes created to analyze the articles included. Every color is associated with each group of codes to facilitate the analysis of corresponding network.

## Results

### State of the Art of End-Of-Life Medical Decisions in Children With Cancer

**Figure 1:**
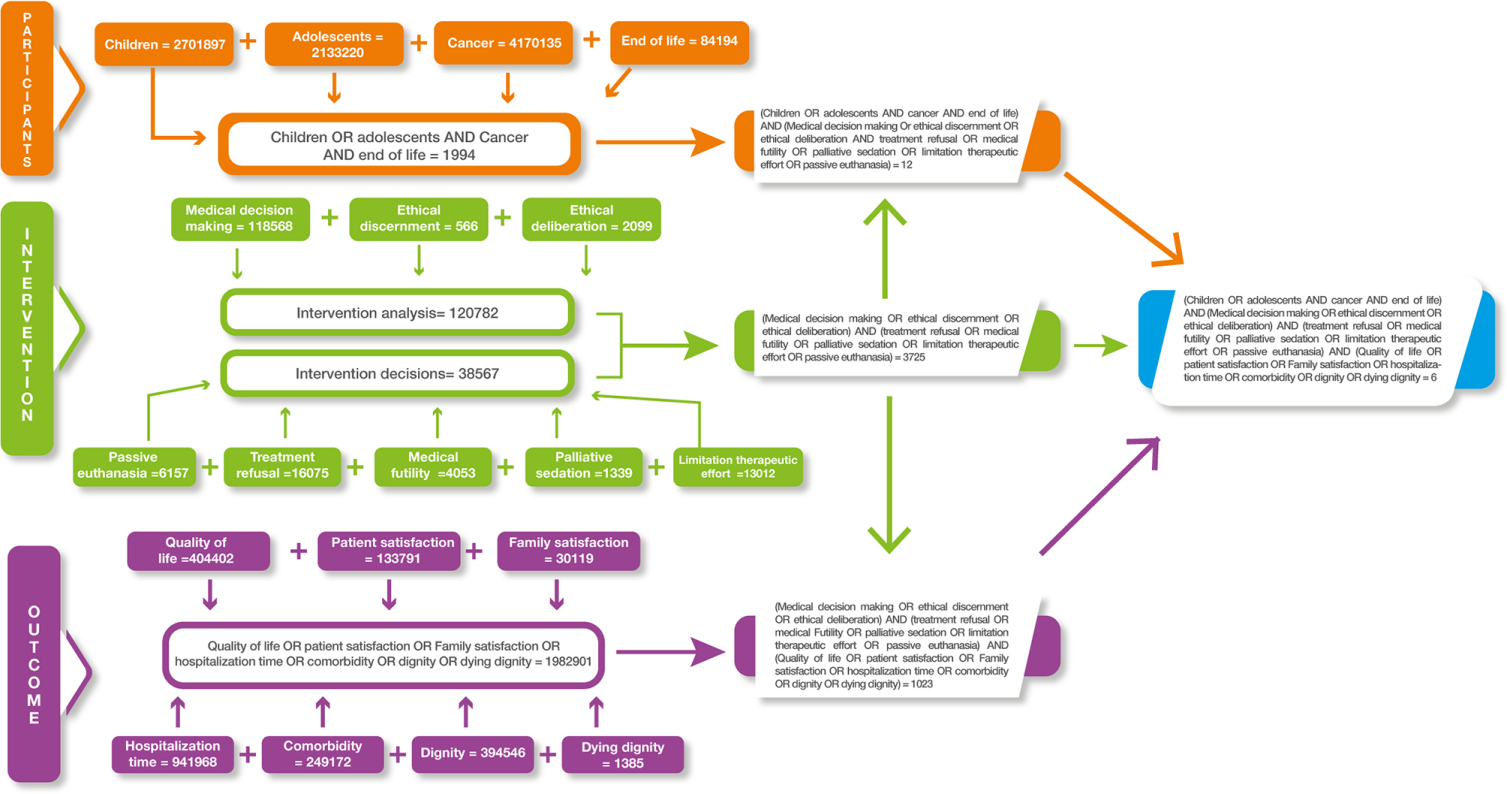
Decision tree used for search in databases. Example of the PUBMED database search.

**Figure 2:**
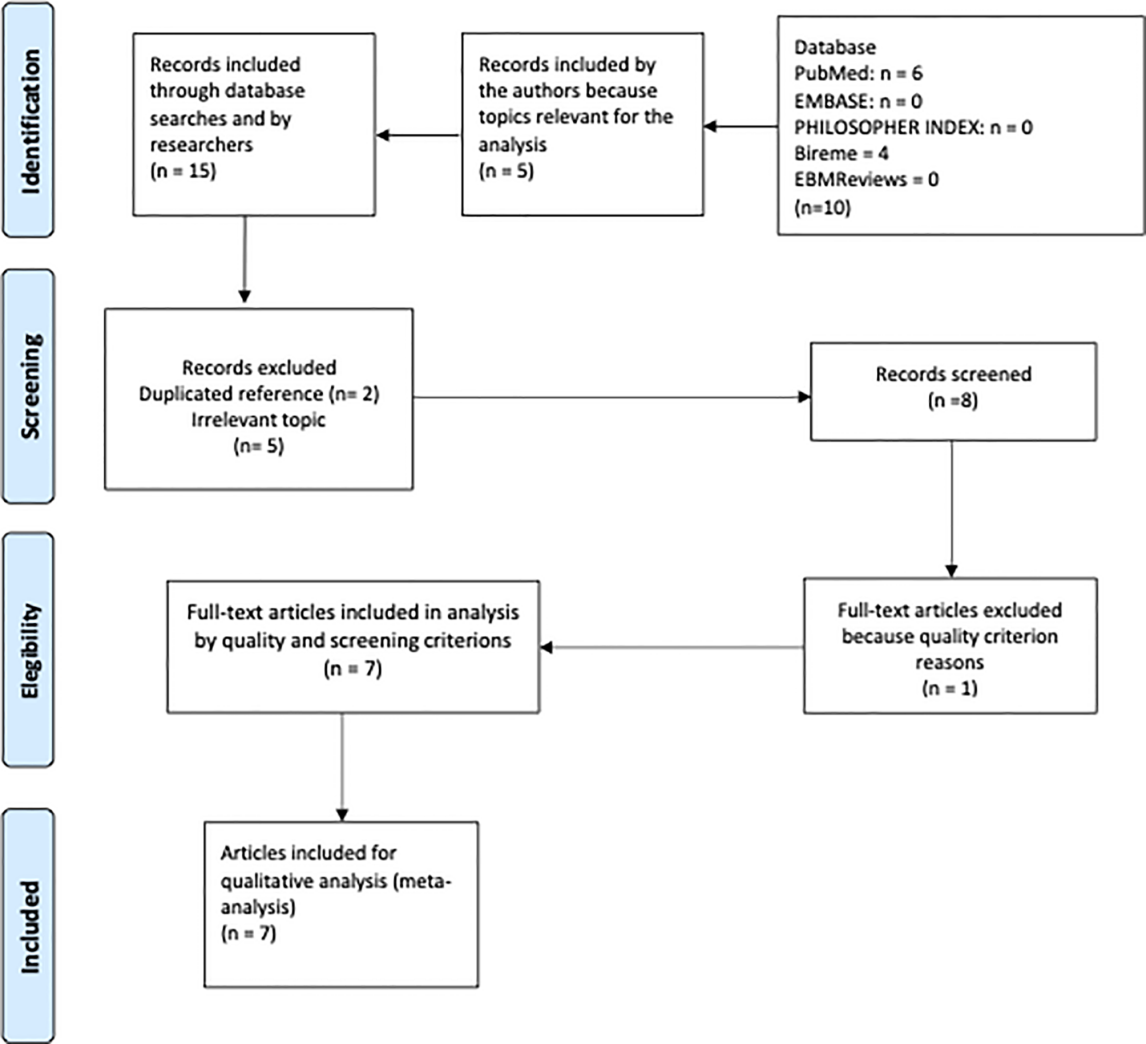
PRISMA flow chart that shows the selection process used to retrieve the final 7 articles.

A systematic review of the literature yielded six articles in PubMed, produced by the National Library of Medicine and four in Bireme (Latin American and Caribbean Center for Information in Health Sciences); no search results were obtained in the other databases. The results of the search strategy in PubMed are shown in **Figure 1**. After the first screening, two studies were excluded because they were duplicate references. During the third screening, the complete article was reviewed methodologically and comprehensively, and two articles met the selection criteria. Due to the epistemic gaps in regard to the topic, five articles were added that complemented the topics of the research question for a more precise analysis of decision-making for pediatric cancer patients. **Figure 2** shows the PRISMA flow chart, which consisted of several screening stages to remove duplicates, topics irrelevant to our research question, and so on.

### Classification and Approach to Bioethical Deliberation in End-Of-Life Decisions for Children With Cancer

Of the seven articles chosen (**Tables 3**, **4**), three were carried out in the United States, one in Italy, one in Mexico, one in Australia, and one in Ireland. The sample or population used in these studies were heterogeneous. Four studies included healthcare personnel, including two in which pediatric oncologists participated, on in which nurses participated, and one in which medical students participated. In the three articles that did not have a sample or population, a systematic review, an articulation of two papers on end-of-life issues and their possible application, and an ethical analysis were carried out. The topics addressed were therapeutic futility and how it influences end-of-life decision-making in two articles, palliative care and advance directives in one article each, and medical decisions at the end of life in general, without developing any in detail, in the final three.

The methodology used was qualitative and quantitative. The analysis was developed through interviews in three articles, clinical studies in two, interpretation of scenarios in one, and document analysis in one.

### Ethical and Legal Gaps in End-Of-Life Decisions for Children With Cancer

The lack of systematic teaching of the ethical and legal aspects of medicine is the fundamental reason for the difficulty in resolving ethical dilemmas at the end of life in the care of children with cancer. Ethical complexity and the availability of parental resources influence the physician when they are faced with the need to decide on life support (17). Other determinants that impact physician decision-making, particularly in the initiation of palliative care, include age, length of practice experience in pediatric oncology, and religious affiliation. Although the physician's religious affiliation has rarely been addressed as a factor influencing their decisions, Lotto found that the sanctity of life and the person's free choice regarding their death (advance directives) in states of diminished consciousness, such as a persistent vegetative state, a minimally conscious state, or brain death, determine the physician's decision-making (19). An important ethical gap is the lack of reference to the axiology of end-of-life decision-making in cancer patients, so much so that only one of the articles analyzed refers to the four principles of bioethics (20).

As a methodological approach for the bioethical analysis, instruments were used to obtain information in five of the studies (two interviews, a survey, an algorithm, and a well-being scale) (14, 15, 17–19) (**Table 3**).

### Main Ethical Dilemmas at the End-Of-Life in Children With Cancer

In cancer patients, the core ethical dilemma concerns the maintenance or withdrawal of supportive care, such as amines, antibiotics, enteral or parenteral nutrition, and mechanical ventilation, because they are considered futile. Armstrong (14) concluded that the articulation of a tool that conceptualizes medical futility will help clinicians with decision-making; the touchstone in this type of patient is the understanding of the decision-making process and the search for palliative care training for a comprehensive assessment of the patient and a better quality of healthcare (15). The triggering vector of a medicine of excellence is the communication between medical staff and parents, strengthened by decisions on end-of-life care, allowing for a more objective assessment of the measures and care required by the child (18). An important ethical gap is the lack of training in communication skills for decision-making personnel (4). There is a predictable correlation between people's moral principles and the negotiation of the acceptance of palliative care that includes, among others, the withdrawal of treatment for patients who request it, which goes beyond the communication process and the understanding of the end-of-life process (19). One challenge in addressing the particular needs of children in these situations is to avoid or decrease the prevalence of medical paternalism (20). This can be achieved through the incorporation of support resources for parents, such as texts or online tools that are based on their child's care decisions (17) (**Table 4**).

### Axiology at the End-Of-Life in Children With Cancer

Value systems (ethical, moral, basic, epistemic, and economic, among others) are at the core of decision-making: with the principled ethical argumentation of his study, Armstrong seeks to avoid medical futility by creating a better definition of this concept in patients at the end of life, considering human dignity and justice as fundamental ethical values, recognizing that decision-making is carried out by the health professional in a shared manner with the family when the risk-benefit ratio is high (14). Specifically in regard to minors, the ethics of medical responsibility together with the doctrine of the mature minor can favor the participation of adolescents with cancer in end-of-life decision-making and thus avoid therapeutic futility (15). Withdrawal or continuation of treatment is another important issue regarding end-of-life care. Physicians and families make shared decisions to maintain or withdraw life-sustaining measures for the patient.

Based on the ethics-of-responsibility argument, the use of a manual and better communication between the medical professional and the family is considered to assist in decision-making through the ethical values of responsibility, commitment, and honesty (17). Autonomy, human rights, and human dignity should be considered by physicians when deciding to continue or withdraw life support, particularly if there is a choice or will previously expressed by the patient; however, moral and ethical principles and their influence on the physician can alter the decision to be made for patients with altered states of consciousness, influenced by the sanctity of life rather than by the free choice previously expressed (19). Sisk study evaluates patient care throughout the disease process, also addressing refusal, abandonment, and adherence to treatment through the doctrine of the mature minor and the principle of justice, under principled argumentation; as an epistemic value, it seeks to increase access to care in adolescents and young adults with greater participation in decision-making (20). From the perspective of principled bioethics and respect for autonomy and the doctrine of the best interests of a child with cancer, it seeks greater participation from the adolescent in decision-making in conjunction with their parents and healthcare professionals throughout the entire process of their disease (16). Few studies have addressed euthanasia and medically assisted suicide in children with cancer. Through an ethics of responsibility argument, in which he finds that pediatric oncologists make end-of-life decisions with little knowledge of medical ethics, palliative care, euthanasia, and other medical decisions, Hilden emphasizes that the ethical values to consider when reflecting on these issues are beneficence, dignity, and human rights (18).

### Axiological Network of End-Of-Life Decisions in a Child With Cancer

For axiological analysis, we used Atlas.tiv.9^®^ software. Each of the articles was examined using this tool based on the value system. The hermeneutic analysis was initially developed with an in-depth reading of the selected articles, in which new codes were included after the interpretation of the actions carried out by physicians in decision-making. These actions were assigned concepts for which definitions were consistent with these codes. Additionally, as part of this analysis, citations and networks of the content of the articles corresponding to each concept were defined. In our analysis, we divided the results into three parts: decisions, principles, and virtues.

The decision dimension included the following issues: refusal of treatment (refusal of therapeutic measures and actions, regardless of their cause), futility (unfortunate situation in which the treatment will not benefit the patient and, therefore, should not be used), limitation of therapeutic effort (elimination of non-beneficial medical treatments), palliative sedation (use of sedative drugs to relieve intractable or refractory pain with decreased patient consciousness), and euthanasia (intentional termination of a person's life by the action of another).

The concepts associated with the group of principled values were respect for autonomy (individual decision-making in healthcare, self-governance free from controlled interference), non-maleficence (obligation not to cause harm to others), beneficence (the act of contributing to the welfare of the individual or humanity), and justice (applying fair and equitable treatment in light of what is owed to individuals).

The concepts associated with the group of virtues were honesty (attribute of accurately revealing the degree of knowledge and ignorance), truthfulness (ability to tell the truth), compassion (willingness to understand, evaluate, and weigh the uniqueness of the patient's disease predicament), integrity (integrating all of the virtues into a whole and judging prudently in order), justice (the habit of rendering what is due to others), and phronesis (virtue of moral thinking, “practical wisdom”).

Six other value groups were created, in addition to the initial three groups. These clusters were decision-oriented and were identified as clinical determinants at the end of life, physician barriers, physician factors, patient and parent factors, patient characteristics, and support tools. All these clusters are included in **Table 5**.

Regarding the clinical determinants at the end of life, four codes were included: awareness, being alive, quality of care at the end of life, and right to die. Medical barriers included group of adolescents and young adults, bad news, lack of communication skills, complexity of sharing information, confusion in decisions, discrepancy in treatment, obstacles to providing care for dying patients, oncologist safety, power relationships, and predictors of medical attitudes and practices. There were 63 medical factor codes, such as beliefs and emotions, the standard of the child's best interests, clinical judgment, communication, ethical training, dishonesty, arrogance, human dignity, moral principles, and paternalism. For the group of parental and patient factors, 26 codes were derived, such as decision quality, physician behavior, shared medical decision-making, parental emotions and beliefs, cultural relationships with decision-making, place and time of death, disagreements between patients and parents, ethnic influences, and unrealistic expectations of cure. The patient group factors were the specific characteristics of cancer in adolescents and young adults, the beliefs and values of this group, child decision-making, child preferences, importance of involving the child, and patient values. From the group of clinical guidelines for decision support, the codes used were the four steps of negotiating principles, advance care planning guidelines, Boston Children's Hospital policy, the Handbook of Care Decisions, the Convention on the Rights of the Child (1989), end-of-life care guidelines, symbolic interactionism, and the Texas advance directives (**Tables 3**, **4**) (14, 16–18, 20).

Three analytical networks were established after the ordering of the codes described above: axiological horizon, foundational values, and medical decisions at the end of life and their relation to virtues.

**Figure 3:**
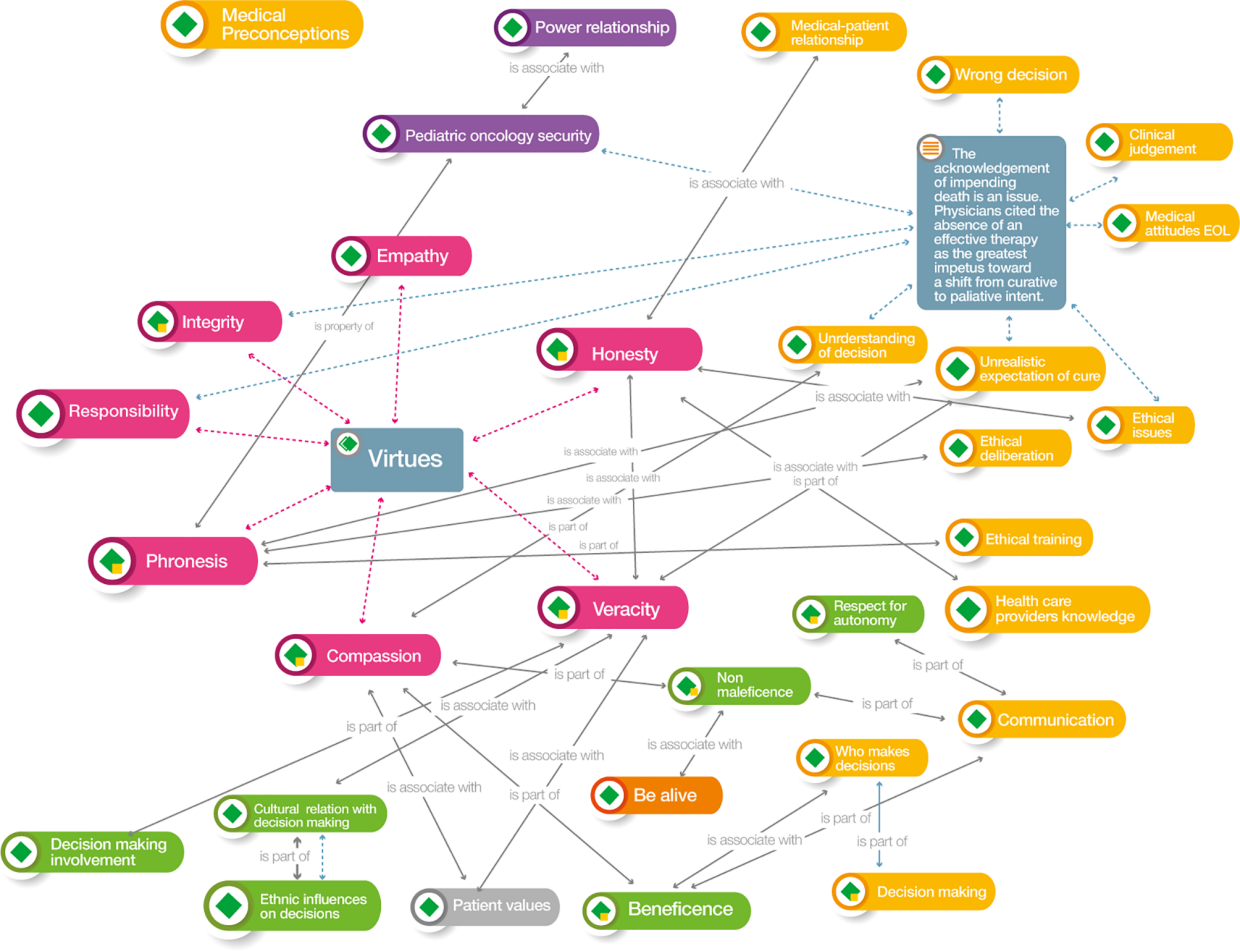
Atlas.ti v.9^®^ Axiological horizon network. Color codes Groups: Red: Medical virtues; Yellow: Medical factors that influence decisions; Dark green: Biomedical ethics principles; Green: Parents-patients’ factors for decision-making; Grey: Patients factors for decision-making; Violet: Medical barriers for decision-making; Orange. Clinical determinants at end-of-life for decision-making.

In the axiological horizon (**Figure 3**), we observed how the physician's virtues influence the factors that determine decision-making, highlighting phronesis and its association with several of these, particularly those related to ethical training and unrealistic expectations of cure. The lack of confidence that pediatric oncologists have in decision-making is considered one of the main barriers, and its manifestation as such is a property of phronesis. The interrelationship between the virtues of compassion and truthfulness with the patient's values allows physicians to seek to make end-of-life care beneficence-oriented, even without the influence of parental determinants. Physician compassion has important bearing on beneficence and non-maleficence—the two *prima facie* principles of principled bioethics most frequently employed by physicians. These principles are integral to physician communication with parents and patients; beneficence is directly involved in decision-making and discussion of who should make decisions. Decisions considered incorrect, clinical judgment, attitudes of patients at the end of life, unrealistic expectations of cure, the physician's understanding of the decision, and the physician's ethical values, the latter associated with their own honesty, influence the shift from curative to palliative therapy. Hilden (18) found that “the acknowledgement of impending death is an issue. Physicians cited the absence of an effective therapy as the greatest impetus toward a shift from curative to palliative intent”. These factors also permeate physician safety and the power relationship established in the physician-patient relationship and are reflected in the values of integrity and responsibility.

The parental factors involved in shared decision-making often relate to the ethnic and cultural influences of the family. These should be guided by the physician through the virtues of truthfulness and honesty, in conjunction with the patient's own values. The physician's preconceptions regarding the definitions of the decisions and their relationship to the virtues and to both the parents' and their own factors, with the subsequent integration of their knowledge of bioethics and its barriers, are present throughout the axiological horizon.

**Figure 4:**
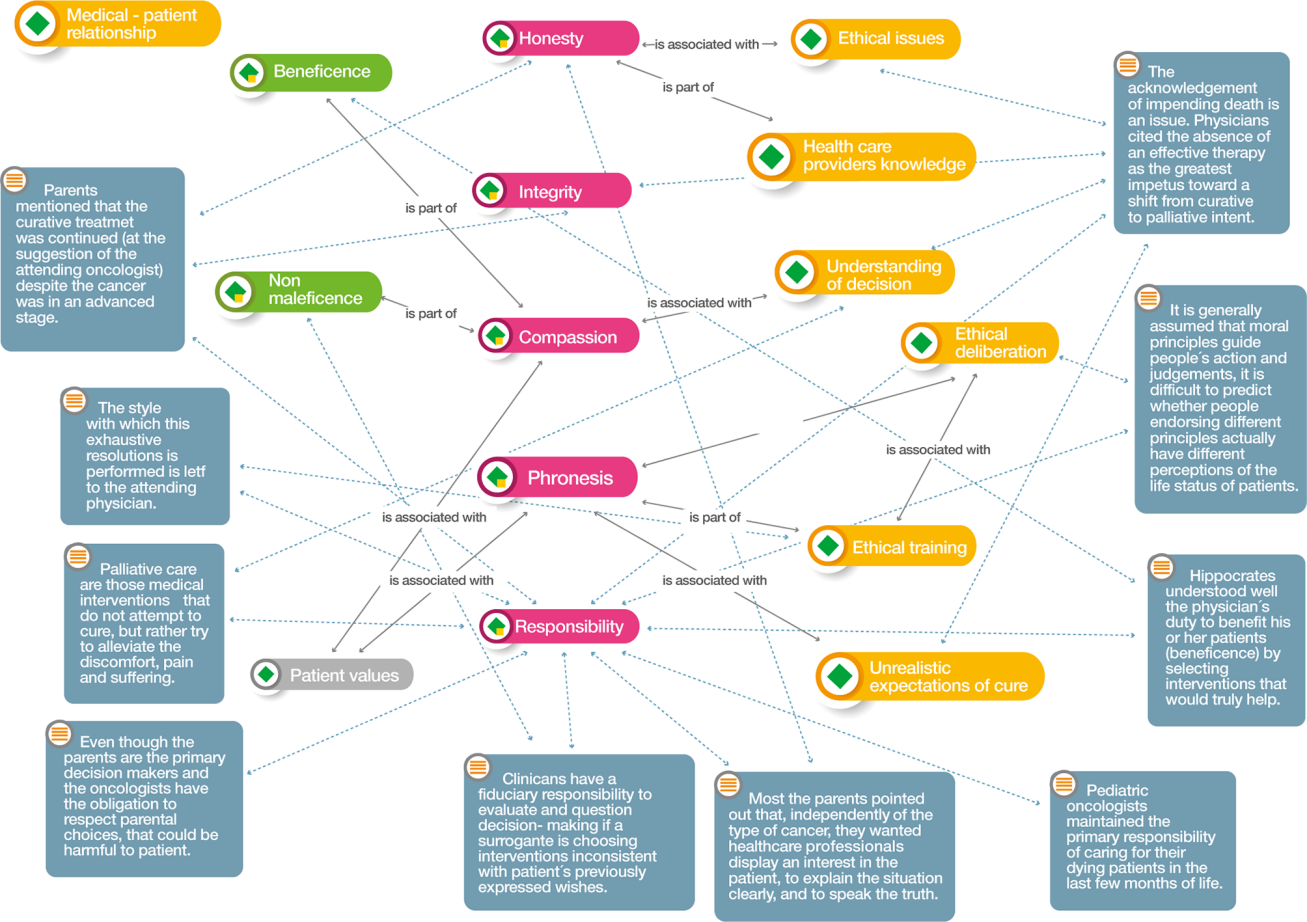
Atlas.ti v.9^®^ Foundational values network. Color codes Groups: Red: Medical virtues; Yellow: Medical factors that influence decisions; Dark green: Biomedical ethics principles; Grey: Patients factors for decision-making.

Foundational values (**Figure 4**). The physician-patient relationship is present at every moment of decision-making and its integration with the foundational values arising from the physician's virtues. The physician's honesty and responsibility combined with knowledge of end-of-life decisions allow them to evaluate the ethical issues involved, particularly the change from curative to palliative therapy. Cicero (15) stated that “palliative care are those medical interventions that do not attempt to cure, but rather try to alleviate the discomfort, pain and suffering”.

Knowing the moment at which the patient should start palliative care, associated with compassion and the patient's values, leads to better judgment (phronesis) in the application of care after the physician's ethical deliberation with their team. Lotto (19) states that “it is generally assumed that moral principles guide people's action and judgements, it is difficult to predict whether people endorsing different principles actually have different perceptions of the life status of patient, “in the same way that Armstrong (14) expresses that “the style with which this exhaustive resolution is performed is left to the attending physician”. Better knowledge of the practical definitions allows the decision to be made when the child's clinical condition—above all, their state of consciousness—is not critical.

The physician's responsibility for decision-making impacts the outcomes obtained by their patients and, as such, should prevail when there is prior knowledge on the part of family members regarding the patient's end-of-life wishes. Sisk (20) mentions that “clinicians have a fiduciary responsibility to evaluate and question decision-making if a surrogate is choosing interventions inconsistent with the patient's previously expressed wishes”.

This intervention will have an impact on the non-administration of therapeutic measures that may cause harm to the child (non-maleficence) and will reflect a compassionate physician. Family members always appreciate that decisions regarding therapeutic measures applied to children are defined by the depth of the physician's involvement with the child. Hilden (18) states that “pediatric oncologists maintained the primary responsibility of caring for their dying patients in the last few months of life,” while according to Cicero (15), “most of the parents pointed out that, independently of the type of cancer, they wanted healthcare professionals display an interest in the patient, to explain the situation clearly, and to speak the truth”.

Non-maleficence, also considered to be among the physician's responsibilities, must be taken into account when parents decide to continue therapeutic measures that may cause harm to the child. In contrast, Cicero (15) found that “even though the parents are the primary decision makers and the oncologist have the obligation to respect parental choices, that could be harmful to the patient.”

In the same way, the compassionate physician always seeks beneficence for their patient through the development of best practices.

Finally, the physician's honesty and mastery of the ethical issues that arise in regard to their patients will allow them to communicate better with the family to avoid the application of unnecessary therapeutic measures. However, in his study, Cicero (15) states that “parents mentioned that the curative treatment was continued (at the suggestion of the attending oncologist) despite the cancer being in an advanced stage”.

**Figure 5:**
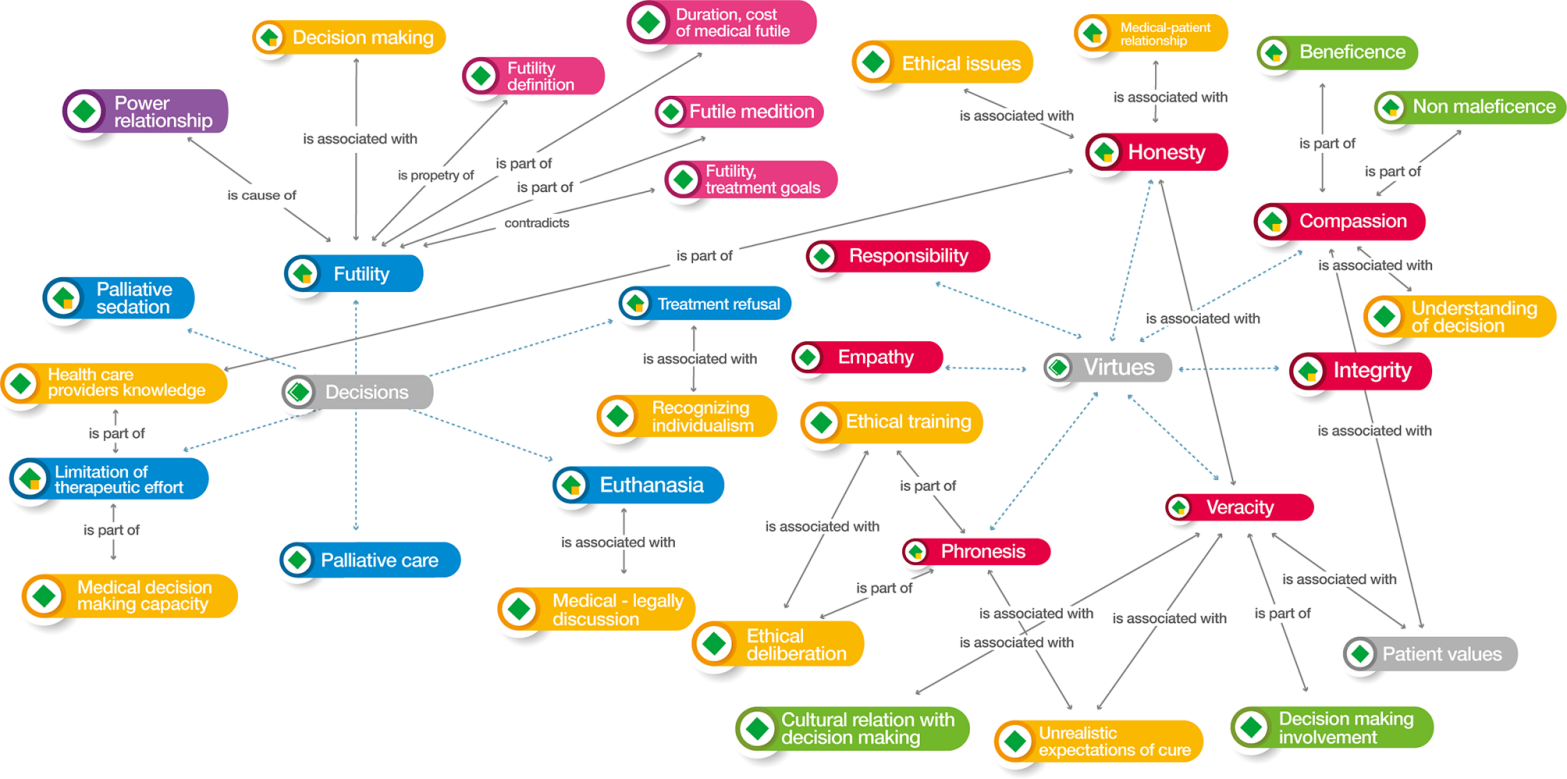
Atlas.ti v.9^®^ Medical decisions at the end of life and their relationship with virtues network. Color codes Groups: Blue: Medical decisions at End-of-Life; Red: Medical virtues; Yellow: Medical factors that influence decisions; Dark green: Biomedical ethics principles; Green: Parents-patients’ factors for decision-making; Grey: Patients factors for decision making; Violet: Medical barriers for decision making; Orange. Clinical determinants at end-of-life for decision-making; Pink: Futility variables.

Medical decisions at the end of life and their relationship with virtues (**Figure 5**). The virtues of physicians who treat children with cancer throughout the disease process are directly involved with the factors that influence end-of-life decision-making. The interaction of phronesis, honesty, truthfulness, and compassion with the ethical aspects of medical practice, especially with the physician's training, the physician-patient relationship, the understanding of the decisions, and the unrealistic expectations of the patient's cure, should allow the physician to relate the results obtained to a better quality of life through the implementation of the principles of beneficence and non-maleficence.

The honesty expressed by the physician, associated with the understanding of medical decisions at the end of life and the care that results from them, favors a greater capacity for choice and allows decisions, especially those related to the withdrawal or maintenance of care, such as the limitation of therapeutic effort, to be reflected in a higher quality of care at this stage. The recognition of the patient's individualism, in the case of adolescents, or, in minors, to the legal responsibility exercised by the parents is associated with another decision that has to do with maintaining or withdrawing therapeutic measures and is defined as refusal of treatment. This decision prevents the continuity of care of the disease process in the child and, in the particular case of cancer, will lead to a relapse of the disease or death of the patient.

Palliative sedation, although among the decisions made by the pediatric oncologist for their patient, is evaluated and applied by pain medicine specialists and palliative care teams in places where these services are available. This leads to the concept being ambiguous or poorly understood by the oncologist and has an impact on the patient's quality of life, which is highly evident in the final months of life.

Therapeutic futility is very difficult to define and, therefore, to recognize, which means that the decisions made by pediatric oncologists for their patients can lead to the application of unnecessary measures for the management of the disease, the symptoms, and the needs of the child. The measurement of futility is also complex because it significantly increases the time and cost of hospitalization. Establishing clear goals of care for the patient involving the child, the family, and the physician avoids therapeutic futility.

Euthanasia is the medical decision at the end of life that yielded the most references at the time of the search; however, its application is directly linked to the medical-legal discussion of the laws applicable in each country. Therefore, bioethical reflection should be aimed at a better understanding of the remaining decisions and the correct application of therapeutic and supportive measures in children; thus, when legislation addresses the issue, there will be sufficient elements for deliberation.

## Discussion

The present study explores the bioethical, axiological, and social factors that have an integral influence on medical decision-making at the end of life for children with cancer, with special emphasis on virtue ethics and its impact on the well-being of patients and their families. This integration of axiological and cross-functional perspectives is groundbreaking in pediatric cancer.

The increase in the cure rates of children with cancer in recent years has made it possible to direct research toward more specific treatments, the reduction of secondary events, and the possible elimination of sequelae that affect quality of life. Likewise, the number of studies focused on the care of patients who cannot be cured has increased. The concepts and definitions of end-of-life medical decisions in these publications are clear; however, their interpretation and application by treating physicians are not always reflected in improved quality of life for patients in their final months of life (21).

The main topics for discussion regarding the results of the systematic review are 1) unsuitable treatments for pediatric cancer patients at the end of life because of 2) a lack of ethical education in the medical trajectory and 3) medical decisions based on personal aspects rather than scientific reasons.

### Unsuitable Treatments for Pediatric Cancer Patients at the End-Of-Life

Up to two-thirds of terminally ill cancer patients undergo invasive procedures, and 60% undergo three or more such procedures; most worryingly, one-fifth of patients who underwent major surgery died within 48 hours of surgery (22). Attempts to create a systematic, comprehensive, and cross-functional method to guide decision-making have not yielded the expected results due to the heterogeneous nature of clinical conditions and pathologies in a child with cancer at the end of life (**Tables 3**, **4**).

The context of end-of-life medical decisions and the focus of the studies are particular to each hospital center; however, there are common bases and themes for analysis. Ofstad et al. proposed a series of categories that can help physicians at the time of the decision, including the following: i) the collection of additional information from the patient beyond that referred to in the file, ii) evaluation of the results of the studies, iii) the definition of the problem, iv) the decision to start, stop, alter, or maintain a drug, v) the intervention of a medical problem related to therapeutic procedures, vi) treatment objectives, vii) legal aspects, and viii) postponement of decisions. With these categories, profiles and maps can be created to provide useful feedback to physicians in their decision-making (23). This criteria proposal for decision-making reasoning can unify the apparent heterogeneity of the articles included in this systematic review.

### Lack of Ethical Education in the Medical Trajectory

On the other side of the coin of technological advances is the dissociation of evidence-based medicine from value-based medicine (EBM-VBM). This concept has as its origins the ethical gaps in the integral bioethical preparation of the physician from their training and throughout their clinical practice. In our review, we did not find that health professionals have formal ethical training; this affirmation was described by other authors. Half of the medical schools in the United States and Canada do not fund ethics curriculum development; moreover, teaching and learning objectives are not entirely clear and are not evaluated (24). The objectives of medical ethics education fall into two main categories: cognitive objectives, which address competencies, and attitudinal objectives, which address virtues (25). Based in the lack of ethical preparation found, we suggest that this is an opportunity for the pediatric oncologists to improve quality of care and obtain training in virtue ethics, which analyzes how the habits that constitute the personal virtues demonstrated by the physician can lead them through wise ethical judgment in medicine to the actual practice of “clinical judgment” (8) and thus promote the good of their patient, as the search for this good determines the obligations and virtues of the health professional, supported by the philosophy of medicine (26).

In contrast to virtue-based medical ethics, in the context of deontology-based medical practice, the advantages of summarizing Beauchamp and Childress's four principles of medical bioethics in a compact system for decision-making involving ethical dilemmas or problems are emphasized (8). This leads to an ethics of minimums for the resolution of medical dilemmas in children with cancer throughout the disease process, particularly at the end of life. We proposed a maximus ethics grounded in the multidimensional approach of our review.

Pediatric oncologists often avoid ethical, legal, and clinical issues in patients for whom there is no realistic chance of cure, partly because of the complexity of the health-disease process and the excess of emotions that are generated. Worry, stress, frustration, fear, mistrust, regret, and even medical exhaustion are present when there is clinical deterioration of the child, even if they are not in the final stage of life (27). **Figures 3** and **5** show the correlation of the virtues, particularly compassion and truthfulness, with decision-making at the end of life and the need to give priority to the physician's honesty in understanding the decisions to be made by the patient at this stage of life.

Related to the previous factors described and confirmed in our analysis, Lyon (28) conducted a longitudinal, randomized, controlled study of advance care planning in adolescents with cancer and how it relates to anxiety, depression, quality of life, advance directives, and spirituality. They analyzed adolescent/family dyads to demonstrate whether it was possible, acceptable, and safe to discuss outcomes reported by the pediatric oncologist and record an advance directive in the patient's record as well as how this influenced spirituality. These interventions were found to significantly alleviate anxiety, increase spirituality, and involve patients in decision-making throughout the disease process.

### Medical Decisions-Making—Personal Rather Than Scientific

#### Accepted Determinants in Medical Decision in Clinical Practice

Generally, the ethics of the pediatric oncologist has bioethical principles at its core, which significantly influences palliative care as the basis on which end-of-life decisions are made for children with cancer. Decision-making based on physician virtue ethics has been only minimally explored, and the multidimensional factors of the disease, such as psychological, emotional, spiritual, and social repercussions, are not always considered in the analysis. Codes created for our analysis considered all these factors.

Physician decisions at the end of life are initially based on the diagnosis, stage, prognosis, and response to treatment of the patient's disease. However, after analysis of the networks (**Figures 3**–**5**), we found that the patient's clinical factors do not always truly define these decisions, mainly due to the lack of integration of value-based medicine with evidence-based medicine. With regard to several different external forces affecting the delivery of high-quality end-of-life care, an “unrealistic expectation of cure” was cited as the most troublesome problem (18).

The complexity of this integration becomes evident when too many aspects outside the patient's measurable disease, such as the family and society, are involved (**Figures 3**–**5** and **Table 4**).

The influence of other factors that are involved in the end of life, such as beliefs, culture, ethnicity, religion, spirituality, experiences, and life expectations, not only of the patients and their families but also of the physicians themselves, have received little research attention (**Figure 5**).

The medical and ethical interpretation of pediatric oncologists regarding the decisions to be made for children in the terminal phase of their disease has led them to initiate palliative care when the patient is in this stage of life and not from the time of diagnosis or from the failure of curative treatment. This leads to the neglect of the patient's basic needs, causing a lower quality of life and the continuation of treatments that are not beneficial to the child (10, 14).

Medical decisions at the end of life in adults are well defined, and their application conforms to previously developed guidelines. Despite this, in a systematic review, McDermott found that, although the professionals responsible for the care of adult cancer patients identify the patient's needs at the end of life, such as symptom control, practical needs, and emotional and spiritual support, in the physician's narrative, attention to these needs is given in a complex and contradictory manner. On the one hand, patient autonomy, choice, and control of needs are privileged, while on the other hand, the interpretation of these needs may be narrow or biased, misinterpreting that a patient is better prepared for death if the display of emotions is contained and controlled (29). This is congruent with our analysis of the factors that influence medical decisions (**Figure 3**).

In the pediatric population, palliative care is the basis for the analysis of these decisions. Few studies have analyzed the transition from palliative care to decisions such as refusal of treatment, palliative sedation, limitation or adequacy of therapeutic effort, and euthanasia in children from the perspective of medical staff. In our analysis, we found that this transition was hindered by deficiencies in communication between the physician and the patient's parents.

#### Communication During the Decision-Making Process

Effective prognostic communication is an essential component of informed decision-making (20). Pediatric oncologists perceive themselves as a component in communicating with dying children and their families and discussing the transition from curative to palliative care. However, researchers who have studied the psychosocial concerns of bereaved parents have reported that families find physician communication vague and confusing, which can lead to anger or a feeling of responsibility for the death of one's child (18). This creates a void of knowledge and reflection on the measures to follow in children who are close to death.

Lack of advance care planning in children has been associated with poor communication, prolonged hospitalizations, poor quality of life, and legal action. The decision to continue or withdraw life-sustaining measures is difficult for parents because the discussion takes place so close to the patient's death, preventing well-reasoned reflection on how this decision will affect the patient and family (**Tables 3**, **4**) (30).

Children need to be involved in making decisions regarding their treatment. This involvement depends on age, their experience of the illness, the type of decision, and the parents' desire for protection from information that may cause suffering (**Figure 3**).

Regardless of their legal standing, young people with illness may have a strong interest in being part of medical-making discussions, and their inclusion and involvement in these discussions is widely supported (20). In general, parents have said that decisions were made within the context of their familial relations and obligations; in some cases, the insistence and preference of the adolescent also influenced the decision (15).

Open and honest communication among the pediatric oncologist, the child with cancer, and their family can help with preparing for the health-disease-death process. Advance decision planning—based on maximal ethics, wherein persons (patient, family, health personnel) and virtues play a central role—in situations of life-threatening diseases should be the standard by which physicians treat their patients (31).

#### Classification of Medical Decisions at the End-Of-Life for Children With Cancer

Because medical decisions at the end of life in pediatric patients with cancer have been analyzed in few articles, including those in our systematic review, we performed an integrative analysis of each of the most frequently considered decisions in clinical practice (**Figure 5**).

##### Palliative Care From a Holistic Perspective

The field of palliative care in pediatric oncology was developed to help children with cancer and their families cope with the physical, psychological, social, and spiritual burdens of cancer and its treatment. The routine integration of palliative care in children, adolescents, and young adults with cancer has led to better outcomes for patients and their families, improved quality of life, and relief of suffering (4).

**Figure 6:**
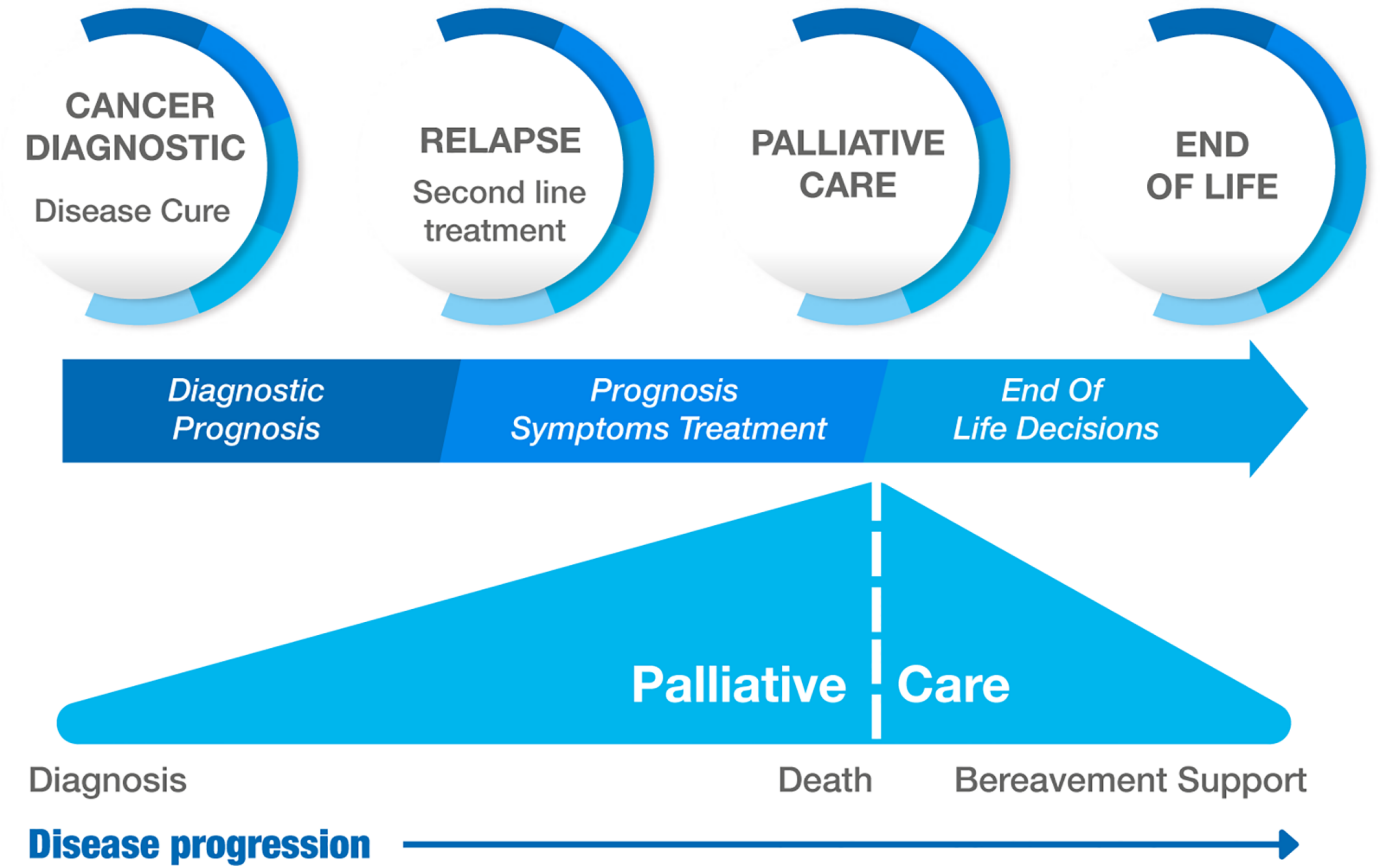
Model of integrative palliative care in children with cancer.

Despite this integration, little is known about the characteristics of the disease trajectories and end-of-life experiences of children who receive palliative care. In addition, many palliative care programs in the United States operate only on weekdays, and a quarter of these are less than five years old (4). The traditional model of integrating palliative care into the disease process of children with cancer has improved care for their needs (**Figure 6**). However, it is necessary to implement a model that also takes into account end-of-life medical decisions that accompany palliative care.

Kaye recently reported the experience of St. Jude Children's Research Hospital (SJCRH) in regard to palliative care with their patients and found that almost 80% of the patients received therapy with experimental protocols, and a quarter underwent bone marrow transplantation prior to death. One-third of the patients received cancer treatment, despite that all had evidence of disease (21).

The accessibility of new targeted cancer therapies, coupled with improved supportive care for these patients, has led to the boundaries among cure, palliation, and death blurring. These ill-defined boundaries also lead to confusion in decision-making on the part of the pediatric oncologist when the child is at the end of life. Guevara found that the most frequent dilemmas reported by a group of pain medicine and palliative care specialists were those related to sedation, home use of opioids, and institutional regulations. These dilemmas were resolved with the practice of virtue medical ethics, among which justice and professional humility stood out, with an ethical core of palliative medicine centered on truthfulness between patients and healthcare providers (32).

##### Palliative Sedation

Many children with cancer die in hospitals each year, some after the difficult decision to withdraw life support and others without reaching intensive care. Regardless of the circumstances leading to intensive care, treating physicians must relieve symptoms throughout the process. Palliative sedation is used to relieve refractory and intractable distress through a reduction of the patient's consciousness; its use in pediatrics also requires parental understanding and consent. Concerns regarding ethical issues persist, particularly in terms of when they are applied and whether they are justified (33).

##### Refusal of Treatment

Refusal is the decision avoid or not accept recommended elective treatment. Pediatric oncologists' attitudes toward refusal of treatment and its approach are highly varied, which leads to much discussion of the topic. Concerns regarding the procedures to be carried out and ethical and legal reflection make resolving the problem difficult. State intervention may be a solution, but when it should be sought is not clear to the physician. When the prognosis of the disease is good, arguing for the best interests of the child define decision-making; when it is bad, parental authority is most important. Caruso found that more than half of the pediatric oncologists surveyed in one study supported refusal of treatment based on the patient's age and chance of cure, more than half supported refusal if the prognosis was poor, and 11% would never support refusal (34).

Seeking court intervention in the resolution of rejection and case perspective for decision-making regarding these patients is recommended in addition to the evaluation of scientific evidence of treatment outcomes, the physician's experience, and clinical judgment. The evolving prognosis also influences decision-making (35). Other factors that influence treatment refusal are socioeconomic status, parental education, religion, ethnic group, and culture (**Figure 3**).

Parents cannot make decisions that cause harm to their children, nor can they refuse treatments that are clearly beneficial. Differences in interpretation among pediatricians are caused by doubts as to whether treatment is ethically obligatory or whether the best interests of the child should be privileged in different scenarios, even if the prognosis of the disease is the same. Differences in philosophical and treatment perception between parents and physicians explain the differences in permissible treatment options, including chemotherapy toxicity. Although guidelines exist for the resolution of treatment refusal, they do not satisfy the complexity of every case, and in the absence of systematic reviews in pediatric oncology, standards of management cannot be easily established (10).

##### Limitation of Therapeutic Effort

Apart from euthanasia—even in countries where it is legislated—the limitation of therapeutic effort is the medical decision at the end of life that causes the most controversy. These controversies arise most frequently when physicians believe that their job is to cure the disease or to maximize the results of medical interventions, often in contradiction to the actual prognosis of the disease. Paradoxically, the physician restricts their involvement in decision-making to provide evidence-based information relevant to the risks and benefits of therapeutic options. Sometimes, the physician is only superficially involved in the relationship with the child and parents, causing loss of trust or errors in assessing the prognosis of the disease. Parental involvement in these decisions represents a very high emotional burden, causing conflicts with the medical team and a continuous search for cancer treatments (35).

Although the issue of treatment limitation is not directly addressed, several of the articles analyzed in the present study reflect on the continuity or withdrawal of advanced life support therapeutic measures (**Tables 3**, **4**) (15, 17–19). In many cases, the lack of ethical preparation of the physicians involved and limited deliberation of these dilemmas leads to therapeutic futility.

##### Therapeutic Futility

This concept refers to treatment and support measures that are continued with the patient and do not provide any benefit; therefore, they should not be used. It is difficult to establish a broader and more precise definition because of the complexity of patient situations. Some classifications strictly use the term futility to refer to the absence of a physiological effect (14). Communication plays the most important role in the decision to continue ineffective treatments, and the pediatric oncologist believes that if they discuss futility with the parents, the parents' state of psychological vulnerability will diminish their ability to understand the fundamental risks of their decision. The personal experience of oncologists, access to second-line protocols, and even the inclusion of the patient in therapeutic trials favor the continuity of treatments in which efficacy and safety are uncertain. In the oncologist's relationship with the patient and their parents, the physician believes that the decision regarding futility is strictly medical; therefore, their role is only to guide the decision based on what they consider beneficial for the patient (**Figure 3**) (15).

Quality of life should be the objective by which futility is measured. The greatest effectiveness of treatment and the most minimal presence of adverse events, even those that are expected, should be the standard for evaluating the continuity of treatment in children with cancer.

##### Euthanasia

However, this medical decision at the end of life is not legislated in most of the world. We believe that it should be analyzed to develop a framework based on a multidimensional approach to all of the medical decisions available. Euthanasia is the intentional termination of life by someone other than the person concerned at the request of the latter. The fundamental conditions established for compliance with laws regarding euthanasia include the requirements of serious and incurable illness and physical or mental suffering (36). In the case of children, legislation is even more limited than for adults. Only the laws on euthanasia in Belgium and the Netherlands provide for its use with minors. Even in those countries, situations will arise for which the law does not provide a solution and in which the palliative care team and physicians are faced with the conflict of ending life and the principle of alleviating suffering (37).

#### Axiological Analysis of End-of-Life Decisions for Children With Cancer

An analysis of medical decisions at the end of life must consider diagnosis, treatment goals, disease prognosis, life and death expectations, values, beliefs, uncertainties, spirituality, and the anticipated consequences of each possible choice considered. In our analysis, all factors were examined considering all the articles, but they never converged in the same health professional.

Although guidelines exist for these purposes and contain consistent ethical principles, there is no guidance on their application in real-life clinical situations. As we observed in our systematic review, children are not considered part of the decision-making process. This issue has been described by a number of authors; however, their publications did not unite the selection criteria. We believe that this argument should be exposed because we think it must be part of this discussion to exalt humanity in medical practice. The SIOP Working Committee on Psychosocial Issues in Pediatric Oncology recognizes children's right to participate in medical decisions in accordance with their developmental level. This guideline aims to encourage physicians to share relevant information regarding their health status in the context of their own culture so that they can actively participate in the decision-making process. The most important points highlight the right of the child to be treated with the best available interventions, the legal responsibility of the parent for the health of their child until they reach the age of majority, the impossibility of children to refuse treatments that have been approved by their parents while they are not of legal age, the right to a full explanation of procedures based on their developmental level of understanding, the right to information of parents from the start and of children as soon as their development allows as well as that this information should not be confusing and unnecessarily complex. Medicine should respect the autonomy of the patient; legal documents should not be oriented only to the protection of the physicians or the institution (38). Clearly, this document seeks to integrate the child into decision-making, but at what point can or should the child be involved in this process? Using interactive interviews, finds that children prefer to be involved in decision-making and clarifies to what degree they are already involved. Most of the children interviewed left decision-making to their parents and physicians, but during the disease process, children become more involved; some do not want to hear about their disease, particularly when they are symptomatic and simply want to feel better. Some prefer sufficient information at the beginning because of the doubts they have, while others want to know the prognosis and what will happen next (31).

The child's participation is limited because they are considered incompetent regarding making decisions because of their age. The process by which children develop awareness and decision-making capacity is different in each case and is subject to the influence of biological, psychological, and social factors. There is no universal agreement regarding the age at which a child should be considered competent to make decisions; children of the same age may have different levels of maturity. To be competent, one needs the mental capacity for decision-making. In some circumstances, such as when one is stressed or under pressure, a bad decision can be made; therefore, the necessary capacity is not sufficient to be considered competent. Through neuroscience, psychology, ethics, and medical practice, four standards for decision-making have been established: the expression of a choice, understanding, reasoning, and judgment (39). Using the MacArthur Competence Assessment Tool (NacCAT), it has been shown that the age of competence for decision-making is 11.2 years; however, in adolescence, there are great changes and developmental leaps in the brain, which can have a contradictory effect on competence (39).

The most frequently mentioned argument for decision-making in minors is the standard of the best interests of the child. However, this is misinterpreted as an absolute rule or duty, which triggers difficult problems to solve when physicians fail to recognize the complexity and changing nature of the desires, emotions, and needs that characterize their relationship with the patient and parents, particularly during the end of life.

One of the issues facing parents of children with terminal cancer is whether to discuss death with their child. Considering the burden of the disease and their responsibility, parents prefer that the possibility of death not be discussed with their children. A child may be aware of the imminence of death, either because of information received or because they are aware of their physical condition and previous experiences. Evidence suggests that terminally ill children benefit from talking about death, but there is no evidence on how parents perceive this communication; it is recommended that communication be open and honest. In a Swedish study of parents who lost a child to cancer, it was reported that parents who discussed death with their children did not regret having done so, while one-third of those who did not discuss it with their children regretted it. It has been suggested that providing accurate information to children regarding the expected course of the disease allows their internal lives and the external world to be congruent, avoiding frustration (40).

The lived experiences of cancer survivors suggest that they should be included in the deliberation of end-of-life decisions. Pousset interviewed 83 adolescent cancer survivors to investigate their attitudes regarding end-of-life decisions and how their lived experiences during the illness influenced their opinions. In terminal situations, up to 90% accepted requests for non-treatment, and 57% to 64% accepted euthanasia, compared to 28% and 11% to 21%, respectively, in non-terminal situations. Adolescents with cancer want to be involved in end-of-life decision-making. They value autonomous decision-making without excluding parents from the process, and previous their experiences influence their attitudes (9).

A “good death” is an unspoken goal to be achieved for children with life-shortening illnesses; thus, the needs of children at the end of life and those of their families must be adequately addressed. While seeking to “do everything possible” with the intention of prolonging life-sustaining treatments, the goal of achieving a good death must be facilitated. The diversity, multiplicity, and complexity of problems in chronic diseases appear to be a journey that evolves over time, involving interactions between various actors and different schemes, with associated trade-offs and impact, ultimately ending in death (41).

The quality of end-of-life care for children with cancer is evaluated differently by pediatric oncologists and parents. Physicians consider that having less pain and not being in the hospital in the final month improves this quality, while parents find that care is improved when there is clear information regarding end-of-life expectations given in a sensitive manner (**Figure 4**). In addition, the timing of discussion regarding medical decisions at the end of life is often late and based on a lack of expectation of recovery, unbearable suffering without a chance of cure, parental request, or expected death in the short term (42). Regarding the reasons for not discussing end-of-life decisions directly with the patient, the emotional aspect is not a reason mentioned.

The association between spiritual and religious factors and patient-reported outcomes and the influence of spiritual and religious constructs on patient-reported anxiety, depressive symptoms, fatigue, and pain are not known. Spiritual problems have been linked to poor health outcomes, particularly those related to mental health. Meaning in life and peace are inversely related to anxiety (43).

The spirituality and religious preferences of pediatric oncologists are not normally considered to be factors in their decision-making. However, for Lucjan Szczepaniak, a physician and chaplain at the University Children's Hospital in Krakow, the pediatrician's responsibility for the health and life of their patient requires that they possess not only professional skills but also an appropriate mental and moral predisposition. He believes that ethical and moral competencies become particularly important when a decision between life and death must be made (44).

## Conclusion

The training and clinical practice of pediatric oncologists has led them to a place where they are perceived as tireless “fighters” for life, moving with warlike language in their daily practice. This is also transmitted to patients and, above all, to their parents. In this context, patients are “warriors” or “fighters” for life, and parents command this struggle. Therefore, the lack of response to treatment is a lost battle, and death is a defeat or failure, something that neither the physician nor the parent can afford. However, here does this “war” leave the patient?

Curative intervention should incorporate sympathetic attention and care on the part of the physician as well as a genuine interest in the child. For adolescents of legal age, there should be guidelines to empower them to make their own decisions with all the rights and privileges of adult patients. For all other children, there should be complete respect for their ability to understand and their desire to participate in decision-making (38).

In this war, each of the participants has different concepts of how to fight, what the results are, and when to know whether the battles are going in the direction of success (healing) or defeat (death). The fighting strategy to win battles can be considered decision-making.

The quality of shared decision-making for children with serious illness depends on parents and physicians who share similar perceptions of the problems and hopes for the children. Disagreements in these perceptions, particularly in regard to the presence and severity of children's symptoms and the possibility of cure, lead to parental concern regarding losing the support of the medical team (45).

With so many factors involved in medical decision-making at the end of life, the oncologist's deliberation must be centered on virtue ethics. There are three reasons why virtue ethics provides a more realistic approach to understanding good medical practice than does rule-based ethics. First, rules or principles are too abstract and general to guide moral action. These rules or principles require interpretation in context, and to achieve this, virtue ethicists emphasize that a good physician must acquire virtues such as perceptiveness and good moral judgment. Second, norms or principles typically set a minimum standard for what is considered good practice and run the risk of fostering an attitude of only meeting these standards. In contrast, virtue-based explanations of medical ethics are “excellence-oriented.” Virtue ethicists are concerned with how the personal virtues demonstrated by the physician in their work can promote the good of the patient to the greatest extent possible (the Greek word for virtue, arête, means excellence). Finally, many authors highlight similarities between wise ethical judgment in medicine and the actual practice of “clinical judgment” (8). In addition, Koetzee (8) concludes that “one of the central questions in medical ethics is how best to understand what characterizes good medical practice or sound medical decisions. Consequentialism in medical ethics sees good practice as ensuring the best outcomes for patients and society, and deontology sees it as practice in conformity with ethical norms or principles. In contrast, virtue ethics sees good practice as practice resulting from the virtuous moral character of the physician. As a distinctive approach to medical ethics, virtue ethics investigates how physicians' good moral character enables them to promote the good for the patient.”

During this systematic review, we did not find evidence regarding how medical values and virtues intervene with other factors, and based on them, what the specific recommendations for decision-making in terminally ill children with cancer are. However, it is clear that the foundations of this decision-making have been addressed and studied, but its application is highly heterogeneous, primarily due to the large number of factors that influence or may influence the interpretation of its application by pediatric oncologists; to the large number of clinical conditions (symptoms, complications, interventions)—emotional, psychological, spiritual, and social—that a child and their family face in the final stage of their life; to the lack of incorporation of these decisions in the legislation of most countries, particularly for the pediatric age group; and finally, to the difficulty of integrating all of these elements from the perspective of the pediatric oncologist and their doctor-patient relationship in seeking the protection of the child and their best interests.

Given the difficulty of clearly establishing which medical criteria are considered by pediatric oncologists when making decisions at the end of life as well as which are personal factors, those of the doctor-patient relationship, and those of the patients and their families that influence the application of these decisions, the researchers propose the realization of a study in which these doctors would be directly questioned regarding the determinants of their decisions.; In such a study, in addition to the causes and consequences of decision-making, the possibility of elaborating a document containing the foundation for the care of children with cancer to be provided under the best available practices would be addressed. All of this should be carried out through virtue ethics and the binomial of evidence-based medicine and value-based medicine.

### Future Directions and Best Practices

**Figure 7:**
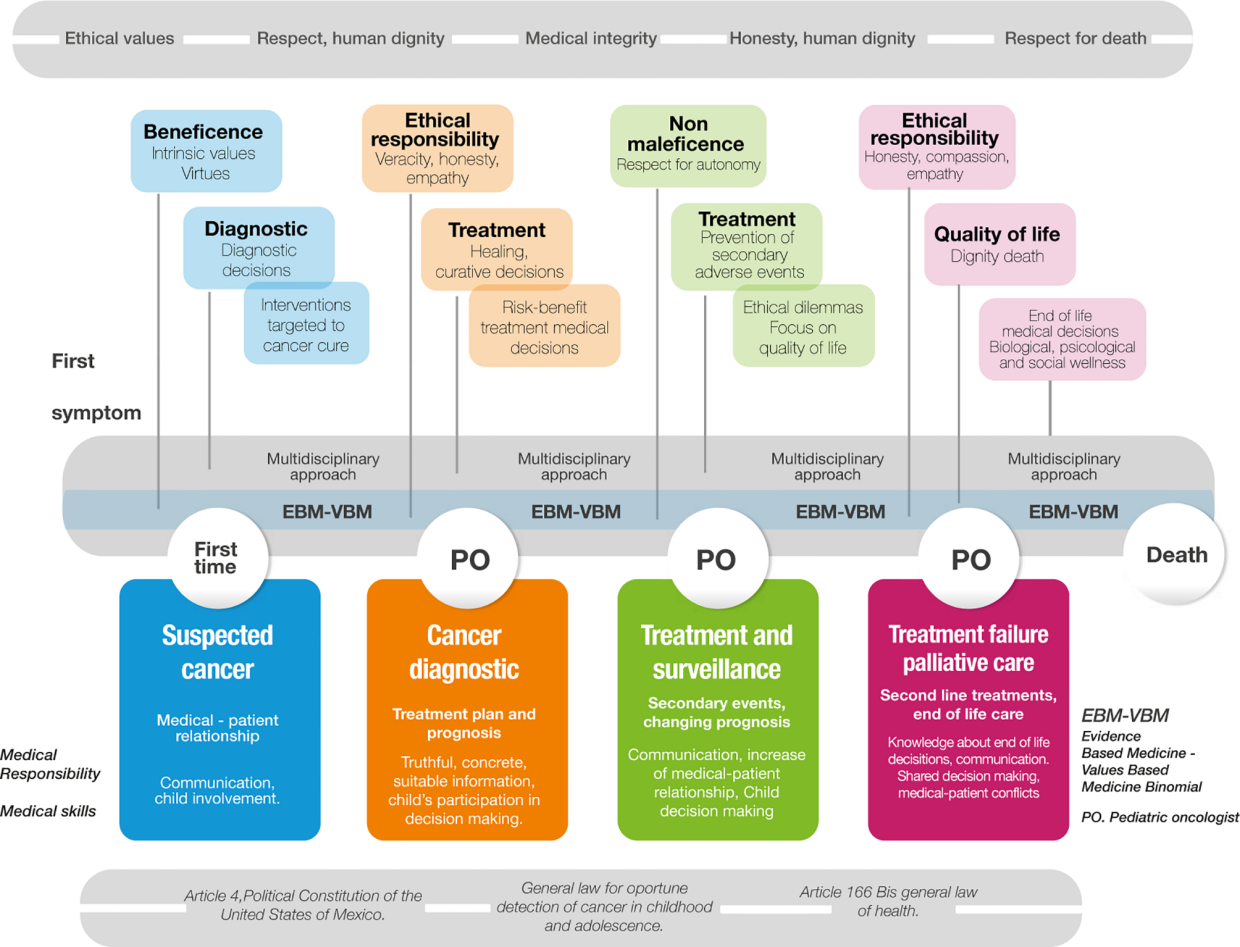
Authors model of multidimensional care for children with cancer based on binomial evidence-based medicine-value-based medicine.

Our cross-functional bioethics group proposes a scheme of integral and multidimensional care for children with cancer during their entire health-disease process, particularly at the end of life, based on virtue ethics and the practice of the binomial evidence-based medicine-value-based medicine (6, 7, 32). This scheme takes into account the legislation in force in our country as well as the application of the skills and abilities acquired for the treatment of the disease and the strengthening of the doctor-patient relationship, favoring communication and the involvement of the child in decision-making (**Figure 7**).

Throughout the entire process, the physician's ethical values are taken into account, such as respect for human dignity, integrity, honesty, and respect for death, as the probability of death is integrated into the mental processes of the physician, patient, and family from the moment the diagnosis is established. Similarly, the multidisciplinary and cross-functional approach, taking into account the practice of the binomial of evidence-based medicine-value-based medicine (EBM-VBM), favors the best decisions during the entire process of the patient's illness. The legislation of our country established in Article 4 of the Political Constitution of the United Mexican States (46) referring to the right to health protection, the General Law for the detection of cancer in children and adolescents (47), and Article 166 Bis of the General Health Law (48) clearly protect children with cancer during the entire process of health and disease.

The doctor-patient relationship, which is the basis for the care of a person facing an illness—in this case, a minor presenting symptom suggestive of cancer—must be established on the basis of trust and communication, with the child or adolescent involved from the beginning. It is at this point that beneficence should be privileged from the practice of the physician's intrinsic virtues and values. To make the best decisions to establish the diagnosis early and in a timely manner, this first approach should already be based on a multidisciplinary approach founded in the practice of the MBE-MBV binomial (**Figure 7**).

Once the diagnosis of cancer has been confirmed, the pediatric oncologist must establish a treatment plan and prognosis for the disease, which is fundamental for decision-making. This prognosis must also be congruent with the technological and human capabilities of the center where the patient is treated. The ethics of responsibility practiced based on the values of truthfulness, honesty, and empathy, establishing a scheme of truthful, concrete, and timely information, and including the patient in the decision-making process, leads the physician to establish the treatment with curative purposes, always evaluating the risks and benefits of their decisions and respecting human dignity.

During cancer treatment, the pediatric oncologist prioritizes intrinsic dignity and respect for autonomy and non-maleficence in the prevention of related adverse events, faces ethical dilemmas related to the patient's quality of life, and is in charge of maintaining communication through a close doctor-patient relationship. During this stage of treatment and follow-up, increased involvement of the minor in decision-making should be encouraged.

Finally, when curative treatments fail, the full integration of palliative care should be encouraged before the possible application of second-line treatments, considering end-of-life care planning. Children are subjects of intense care/treatment at the end of life, which is complicated by their inability to choose or decide on their own needs, leading to disparities between the patient's goals and those of their family (49). At this stage of the patient's life, the physician must prioritize the ethics of responsibility, favoring honesty, compassion, and empathy as virtues and values, with the intention of providing the best quality of life for the patient through knowledge and application of end-of-life decisions based on the biological, psychological, and social well-being of the child and their family. Moreover, in this final phase of life, conflicts may arise between the physician and the patient and family members regarding shared decision-making; however, we must always seek to dignify the imminent death of the patient.

“There is a moral core to healing in all societies that I take to be the central purpose of medicine. That structure is luminously revealed by the experience of illness and by the demands made on the patient-doctor relationship; it is clouded over by a narrow examination of the nontherapeutic aspects of healing. The accounts in this book reveal that the experience and meanings of illness are at the center of clinical practice. The purpose of medicine is both control of disease processes and care for the illness experience. Nowhere is this clearer than in the relationship of the chronically ill to their medical system: for them, the control of disease is by definition limited; care for the life problems created by disorder is the chief issue” (50).

## Data Availability Statement

The original contributions presented in the study are included in the article, further inquiries can be directed to the corresponding authors.

## Author Contributions

LJ-V and MA-B conceived and designed the study. LJ-V, MA-B, and MZ-T performed the systematic research and/or bioethical meta-analysis and analyzed the data. LJ-V, MA-B, and MZ-T wrote the paper and contributed to helpful discussions. All authors contributed to the article and approved the submitted version.

## Conflict of Interest

The authors declare that the research was conducted in the absence of any commercial or financial relationships that could be construed as a potential conflict of interest.

## Publisher’s Note

All claims expressed in this article are solely those of the authors and do not necessarily represent those of their affiliated organizations, or those of the publisher, the editors and the reviewers. Any product that may be evaluated in this article, or claim that may be made by its manufacturer, is not guaranteed or endorsed by the publisher.
